# MPK6‐mediated HY5 phosphorylation regulates light‐induced anthocyanin accumulation in apple fruit

**DOI:** 10.1111/pbi.13941

**Published:** 2022-10-27

**Authors:** Yifan Xing, Wenjing Sun, Yuying Sun, Jialin Li, Jie Zhang, Ting Wu, Tingting Song, Yuncong Yao, Ji Tian

**Affiliations:** ^1^ Beijing Advanced Innovation Center for Tree Breeding by Molecular Design Beijing University of Agriculture Beijing China; ^2^ Plant Science and Technology College Beijing University of Agriculture Beijing China; ^3^ College of Horticulture China Agricultural University Beijing China

**Keywords:** MPK6, phosphorylation, HY5, light, anthocyanin, apple fruit

## Abstract

Light is known to regulate anthocyanin pigment biosynthesis in plants on several levels, but the significance of protein phosphorylation in light‐induced anthocyanin accumulation needs further investigation. In this study, we investigated the dynamics of the apple fruit phosphoproteome in response to light, using high‐performance liquid chromatography–tandem mass spectrometry analysis. Among the differentially phosphorylated proteins, the bZIP (basic leucine zipper) transcription factor, HY5, which has been identified as an anthocyanin regulator, was rapidly activated by light treatment of the fruit. We hypothesized that phosphorylated MdHY5 may play a role in light‐induced anthocyanin accumulation of apple fruit. Protein interaction and phosphorylation assays showed that mitogen‐activated protein kinase MdMPK6 directly interacted with, and activated, MdHY5 via phosphorylation under light conditions, thereby increasing its stability. Consistent with this finding, the suppression of the mitogen‐activated protein kinase genes *MdMPK6* or *MdHY5* resulted in an inhibition of anthocyanin accumulation, and further showed that light‐induced anthocyanin accumulation is dependent on MdMPK6 kinase activity, and is required for maximum MdHY5 activity. Under light conditions, active MdMPK6 phosphorylated MdHY5 leading to accumulation of phospho‐MdHY5, which enhanced the binding of MdHY5 to its target anthocyanin related genes in fruit. Our findings reveal an MdMPK6–MdHY5 phosphorylation pathway in light‐induced anthocyanin accumulation, providing new insights into the regulation of light‐induced anthocyanin biosynthesis in apple fruit at both the transcriptional and post‐translational levels.

## Introduction

Anthocyanin pigments are a class of secondary metabolites and are responsible for the red, purple and blue colours of various organs in a wide range of plant species (Allan *et al*., 2008; Grotewold, 2006; Koes *et al*., 2005). Anthocyanins not only have multiple physiological functions in plants, such as aiding pollination and seed dispersal and adaption to adverse environmental conditions (Winkel‐Shirley, 2001), but are also natural antioxidants with benefits as dietary components for human health, such as reducing cardiovascular disease and liver damage (Bondonno *et al*., 2019; Czank *et al*., 2013).

More than two decades of studies have revealed that anthocyanins are synthesized through the flavonoid pathway and that the associated biosynthetic genes are coordinately regulated by a MYB‐bHLH‐WD40/WDR (MBW) transcription factor (TF) regulatory complex (Albert *et al*., 2011; Ballester *et al*., 2010; Koes *et al*., 2005; Ramsay and Glover, 2005). The central role of the members of this complex in regulating anthocyanin biosynthesis has been documented in a broad range of land plant species (Allan *et al*., 2008; Koes *et al*., 2005; Ramsay and Glover, 2005). In apple (*Malus domestica*), three MYB TFs, namely, MdMYB1, MdMYB10, and MdMYBA, have been functionally characterized and shown to directly regulate the expression of anthocyanin biosynthetic genes (Ban *et al*., 2007; Espley *et al*., 2007; Takos *et al*., 2006). MdbHLH3, a low‐temperature‐induced bHLH TF from apple, interacts with MdMYB1 and induces its expression and that of anthocyanin biosynthesis genes, thereby promoting anthocyanin accumulation (Xie *et al*., 2012). Recent studies showed that other TF families are also involved in anthocyanin synthesis, including members of the bZIP, NAC, Homo domain‐leucine zipper (HD–ZIP), WRKY, and Ethylene‐Response Factor (ERF) families, indicating a broad level of regulation at the transcriptional level (An *et al*., 2017, 2018a,b, 2019a,b; Liu *et al*., 2019; Sun *et al*., 2019).

In addition to temperature (Ubi *et al*., 2006; Xie *et al*., 2012), environmental factors such as light (Bai *et al*., 2019a,b; Fang *et al*., 2019) and nutrient availability (Jiang *et al*., 2007; Kadir and Sorkel, 2005; Schaberg *et al*., 2003; Sinilal *et al*., 2011), as well as exogenous hormones (An *et al*., 2018a,b; Wang *et al*., 2019), can influence anthocyanin biosynthesis by altering the transcription of anthocyanin‐related genes. Light, an important signal for plant growth and development, has been particularly closely associated with inducing anthocyanin accumulation (Li *et al*., 2011). In apple, MdCOP1 (CONSTITUTIVE PHOTOMORPHOGENIC 1), a specific E3 ubiquitin ligase for MYB1, enhances the expression of *MdMYB1* to regulate anthocyanin biosynthesis under light conditions, and promotes the degradation of MdMYB1 via the ubiquitin pathway in the nucleus under dark conditions (Li *et al*., 2012, 2018). The bZIP transcription factor HY5 (ELONGATED HYPOCOTYL 5), acts as a central transcriptional regulator in light signalling by mediating photoreceptor responses to promote photomorphogenesis (Gangappa and Botto, 2016). Recent studies also showed that HY5 is a positive regulator of anthocyanin accumulation through direct binding to the promoters of genes involved in anthocyanin biosynthesis. In tomato (*Solanum lycopersicum* L.), SlHY5 directly binds to the G‐box and ACE motifs in the promoters of anthocyanin biosynthesis genes, such as *CHS1*, *CHS2*, and *DFR* and thereby regulates anthocyanin biosynthesis in fruit (Liu *et al*., 2018). In addition, MdHY5 functions upstream of light‐induced MYB transcription and directly binds to the G‐box motif of the *MdMYB10* promoter to induce its expression, thereby also regulating light‐induced anthocyanin biosynthesis (An *et al*., 2017). Finally, MdBBX20 encodes a B‐box zinc finger protein that promotes anthocyanin accumulation synergistically with MdHY5 under light conditions (Fang *et al*., 2019). Despite all this knowledge, the mechanism of transcriptional HY5 regulation during light‐induced anthocyanin accumulation is still not well understood.

In addition to transcriptional regulation, post‐translational processes are critical factors for the output of gene expression. Protein phosphorylation is one of the most abundant and best understood post‐translational modifications and affects signal transduction pathways by modulating protein activity and protein–protein interactions (Mithoe and Menke, 2011). Indeed, phosphorylation has been estimated to affect one‐third of eukaryotic proteins (Qeli and Ahrens, 2010). Several studies have shown that protein phosphorylation is a part of light‐induced developmental processes. For example, *Arabidopsis thaliana* MPK6 (mitogen‐activated protein kinase 6) phosphorylates PIF3 (phytochrome‐interacting factor 3) to regulate red light‐induced cotyledon opening during seedling photomorphogenesis (Xin *et al*., 2018), and the *A. thaliana* MKK3‐MPK6 cascade has been shown to be activated by blue light to regulate hypocotyl growth through phosphorylation of MYC2 (Sethi *et al*., 2014). Furthermore, our recent study showed that MdMPK4 can be activated in response to light, and that it phosphorylates MdMYB1 to increase its stability and promote anthocyanin accumulation (Yang *et al*., 2021), suggesting that MAPK‐mediated protein phosphorylation contributes to anthocyanin accumulation.

A previous study showed that HY5 is also regulated by phosphorylation at its serine 36 residue, although the kinase responsible for this phosphorylation remains unknown (Hardtke *et al*., 2000). Furthermore, a recent study showed that SPA1 (Suppressor of phytochrome A‐105) can directly phosphorylate HY5 and fine tune its stability and activity when regulating photomorphogenesis (Wang *et al*., 2021a). Taking these studies into consideration, we reasoned that HY5 phosphorylation may also be important for anthocyanin biosynthesis in apple fruit. In this study, we employed liquid chromatography (LC)‐mass spectrometry (MS)/MS based label‐free proteomics to show that HY5 was rapidly activated by light treatment of the fruit, and that MdMPK6 interacts with, and phosphorylates MdHY5. MdMPK6 functions to maintain the phosphorylation status of MdHY5, thereby preventing its ubiquitination by MdCOP1, leading to increased expression of anthocyanin related genes, elevated MdHY5 TF activity and increased anthocyanin accumulation.

## Results

### Immunodetection of apple peel protein phosphorylation

**Figure 1:**
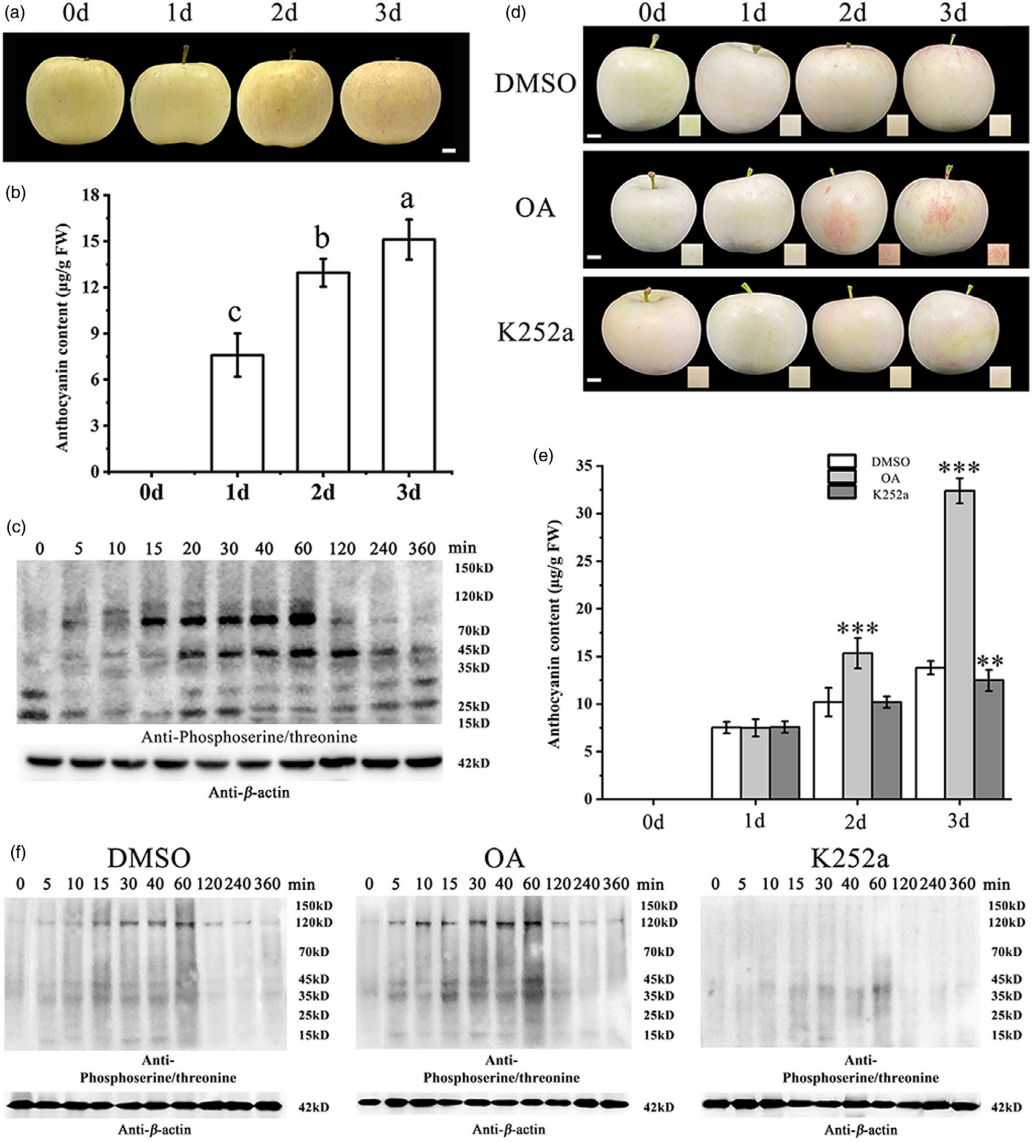
Protein phosphorylation analysis in light treated apple fruits. (a) ‘Red Fuji’ apple fruit phenotype after light treatments. Scale bars = 1 cm. (b) Anthocyanin content of apple fruit (μg/g fresh weight). (c) Protein phosphorylation was analysed by immunoblotting with phosphoserine and phosphothreonine antibodies after different light treatments. (d) Apple fruit phenotypes after treatment with a kinase inhibitor (K252a) or a phosphatase inhibitor (OA). Un‐treated apple fruit at the indicated time points were used as a control. (e) Anthocyanin content in treated apple peels (μg/g fresh weight). (f) Protein phosphorylation was analysed by immunoblotting with phosphoserine and phosphothreonine antibodies after OA and K252a treatments. Asterisks indicate significant differences compared to the DMSO treatment (Student's *t*‐test, **P < 0.01, ***P < 0.001).

In apple peels, light is considered to be a key factor in the regulation of anthocyanin synthesis (Espley *et al*., 2007). In our study, apples were bagged during growth and then transferred from the dark conditions to an incubator for light treatments. Apple peels showed visible signs of anthocyanin accumulation after 3 days of continuous light treatment (Figure 1a,b). To determine whether protein phosphorylation is involved in light‐induced apple pigmentation, apple (*Malus domestica* ‘Red Fuji’) fruit were treated with light for 0, 5, 10, 15, 30, 40, 60, 120, 240, and 360 min and then protein extracts from the corresponding peels were used for immunoblot analyses with phosphoserine and phosphothreonine antibodies. The intensity of the phosphorylation bands peaked in the 60 min sample, and then gradually decreased until the 360 min sample. Phosphorylated polypeptides of 23, 45, and 90 kDa were observed after 60 min light treatment, while only 23 and 45 kDa peptides were detected in response to light treatment for 360 min (Figure 1c).

To further confirm that the MAPK cascade is involved in fruit coloration, apple fruits were treated with a kinase inhibitor (K252a) or a phosphatase inhibitor (OA, okadaic acid). The OA treatment promoted anthocyanin accumulation and red coloration around the spraying site, whereas K252a treatment inhibited fruit coloration (Figure 1d). High‐performance liquid chromatography (HPLC) analysis of anthocyanin contents further confirmed the phenotypic observation (Figure 1e). Immunoblotting showed that K252a treatment caused a decrease in phosphorylation, while OA treatment caused a substantial increase in the signal of the phosphorylated bands, compared to the control (DMSO treatment; Figure 1f). These results indicated that light‐induced anthocyanin accumulation in apple fruit is controlled by protein phosphorylation.

### Label‐free phosphorylation mass spectrometry identification of phosphorylated apple peel proteins following light treatment

**Figure 2:**
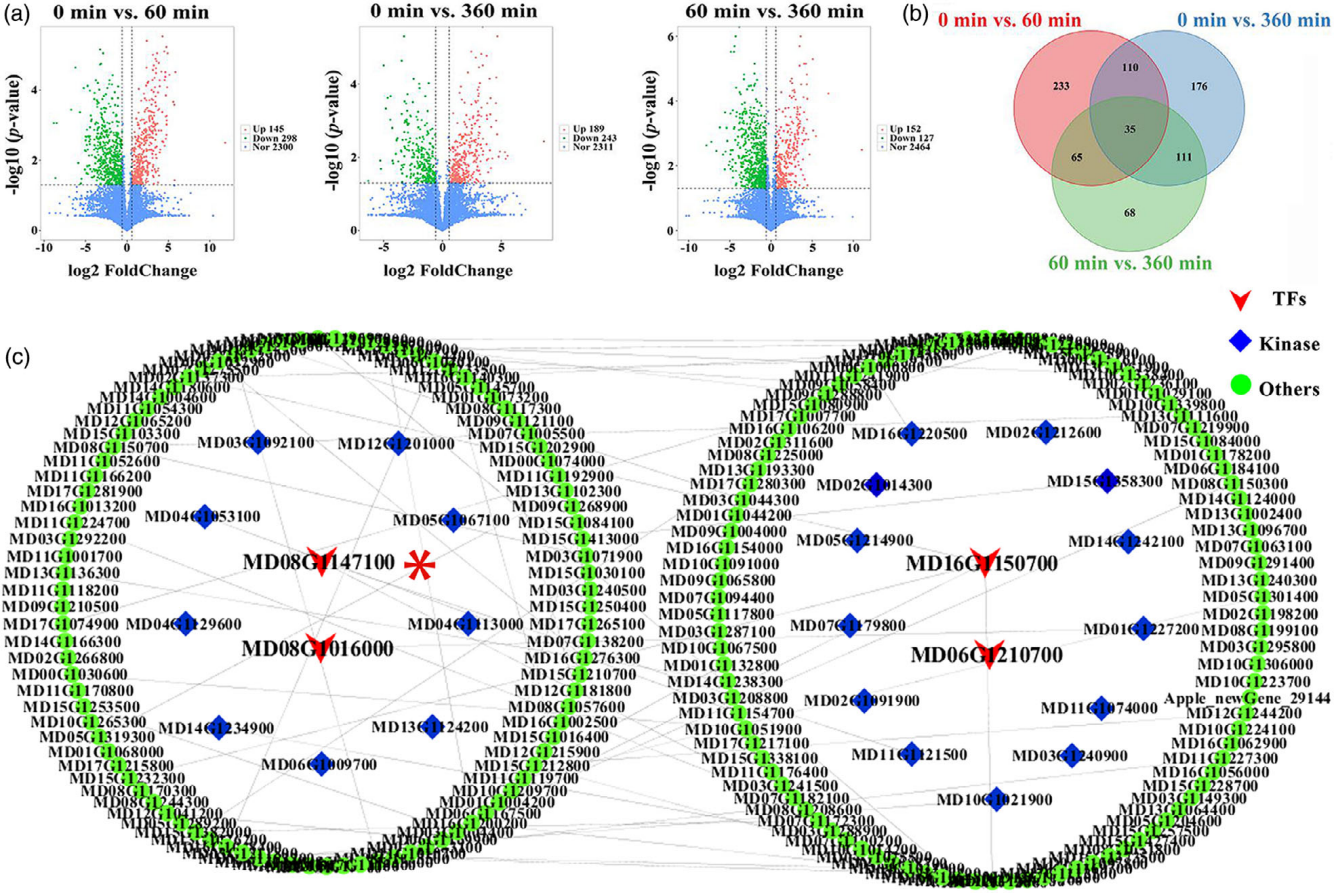
Analysis of mass spectrometer (MS) data. (a) Volcano plot showing the differentially phosphorylated proteins (DPPs) in red and green. The X‐axis represents the fold change in 0 min vs. 60 min, 0 min vs. 360 min and 60 min vs. 360 min comparisons (on a log2 scale). The *Y*‐axis represents the negative −log10 transformed average FPKM (Fragments Per Kilobase per Million) values. (b) DPP Venn diagrams showing different comparisons (0 min vs. 60 min, 0 min vs. 360 min and 60 min vs. 360 min). (c) Protein–protein interaction (PPI) network of DPPs performed using STRING. Transcription factors (TFs) are labelled in red, kinases are labelled in blue and other proteins are labelled in green. Detailed protein information is provided in Table S3. Asterisk (*) represent MdHY5 in the (c).

We profiled phosphoproteome changes in peels from apples that had been light treated for 0, 60 and 360 min. Phosphopeptides were enriched using TiO_2_ affinity and identified in triplicate biological replicates by MS. A total of 3403 proteins and 7420 peptides were identified (Table S1), and 5003 phosphorylated peptides with 8634 phosphorylation sites, corresponding to 2901 phosphorylated proteins were recognized (Table S2). Analysis of the phosphorylated proteins revealed a significant difference in phosphorylation after light treatment. Furthermore, the number of phosphorylated proteins after a 60 min treatment was significantly higher than after a 360 min light treatment, indicating that the phosphorylation response occurred within a few hours. Using a log2(fold‐change) > 1 and false discovery rate (FDR) < 0.05 cutoff, we identified 145 differentially phosphorylated proteins (DPPs) as being higher abundance up‐regulated and 298 as being lower abundance in the 60 min sample compared with the 0 min sample. The total number of DPPs in 0 min vs. 360 min was 432, with 189 up‐regulated and 243 down‐regulated. For the 60 min vs. 360 min comparison, we found 279 DPPs, with 152 up‐regulated, and 127 down‐regulated (Figure 2a), while there were 35 common DPPs between 0 min vs. 60 min, 0 min vs. 360 min and 60 min vs. 360 min, with 15 being up‐regulated and 20 being down‐regulated (Figure 2b). A total 798 DPPs were used to perform a PPI (protein–protein interaction) network analysis to investigate their relationships (Figure 2c). The confidence score was set at the highest level (≥0.900), and using the STRING database combined with Cytoscape, a complex PPI network was produced involving the identified phosphoproteins (Table S3). Notably, the bZIP protein MdHY5 was found in the PPI network. MdHY5 has previously been shown to regulate light‐induced anthocyanin biosynthesis in apple fruit, and to bind to the promoters of anthocyanin biosynthesis genes *MdCHI* and *MdUFGT* and the anthocyanin biosynthesis regulator *MdMYB1* (An *et al*., 2017). We therefore targeted *MdHY5* for further analysis.

### MdMPK6 proteins interact with MdHY5

**Figure 3:**
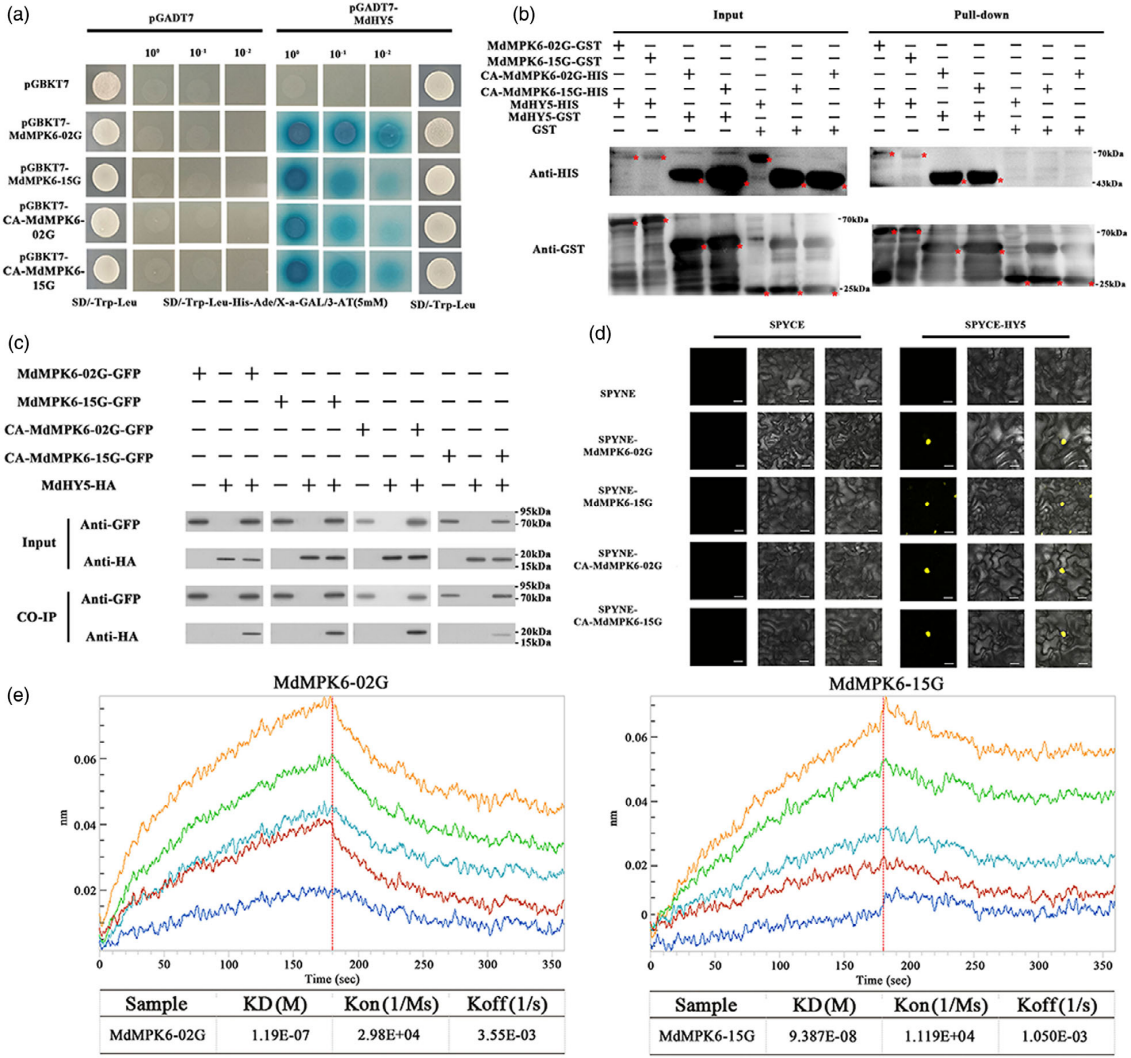
MdMPK6 and MdHY5 interactions. (a) Constitutively activated MdMPK6 proteins (CA‐MdMPK6) and native MdMPK6 proteins, interact with MdHY5 in yeast as indicated by the blue colour. The empty pGADT7 prey vector was used as a negative control. (b) *In vitro* pull‐down assay showing the interaction of MdMPK6 with MdHY5. GST‐MdMPK6‐02G or GST‐MdMPK4‐15G was immobilized on glutathione sepharose beads, incubated with MdHY5‐HIS and subjected to immunoblot analysis with an anti‐GST or anti‐HIS antibody. (c) MdMPK6 proteins associate with MdHY5 in *Nicotiana benthamiana* leaves. Proteins were extracted from tobacco leaves co‐transfected with CA‐MdMPK6‐GFP and MdHY5‐HA proteins and immunoprecipitated using anti‐HA agarose beads. Co‐immunoprecipitated MdHY5‐HA was detected using an anti‐HA antibody. (d) Bimolecular fluorescence complementation (BiFC) assay in tobacco leaves. Yellow indicates a positive interaction. Scale bar = 25 μm. (e) A biolayer interferometry (BLI) assay showing the association and dissociation curves of MdMPK6‐02G and MdMPK6‐15G with MdHY5.

In our previous study, we found that light treatment led to rapid phosphorylation of MdMPK3, MdMPK4, and MdMPK6 (Yang *et al*., 2021). Here, yeast two hybrid (Y2H) assays were used to test the interaction between MdMPK3 proteins (MdMPK3‐03G, MD03G1108500 on chromosome 3 and MdMPK3‐11G, MD11G1121500 on chromosome 11), MdMPK4 proteins (MdMPK4‐06G, MD06G1089500 on chromosome 6 and MdMPK4‐14G, MD14G1110400 on chromosome 14), MdMPK6 proteins (MdMPK6‐02G, MD02G1004000 on chromosome 2 and MdMPK6‐15G, MD15G1147300 on chromosome 15) with MdHY5. Constitutively active forms (CA‐MdMPK3, CA‐MdMPK4, and CA‐MdMPK6), which results from two mutations in each of the conserved domains of MdMPK3 (E192G; E196A), MdMPK4 (D198/200G; E202/204A), and MdMPK6 (D224/227G; E228/231A) were then used as baits. We observed that MdMPK6‐02G, MdMPK6‐15G, CA‐MdMPK6‐02G, and CA‐MdMPK6‐15G specifically interacted with MdHY5 (Figure 3a, Figures S1,S2).

Next, we performed a pull–down assay to confirm the interactions between MdMPK6 and MdHY5 *in vitro*. We generated recombinant MdHY5, MdMPK6‐02G, and MdMPK6‐15G each with a GST (glutathione S‐transferase) tag, then incubated the tagged proteins with recombinant HIS‐tagged CA‐MdMPK6‐02G‐6×HIS, CA‐MdMPK6‐15G‐6×HIS, MdHY5‐6×HIS or GST alone. GST‐MdHY5 was pulled down by CA‐MdMPK6‐02G‐6×HIS and CA‐MdMPK6‐15G‐6×HIS, GST‐MdMPK6‐02G, and GST‐MdMPK6‐15G were pulled down by MdHY5‐6×HIS but not by GST alone, indicating that MdHY5 directly binds to MdMPK6 *in vitro* (Figure 3b). To validate the interaction between the MdMPK6 proteins and MdHY5 *in vivo*, we performed a coimmunoprecipitation (Co‐IP) assay using *Nicotiana benthamiana* leaves. *35S::HA‐MdHY5* and *35S::MdMPK6‐02G‐GFP, 35S::MdMPK6‐15G‐GFP, 35S*::CA‐*MdMPK6‐02G‐GFP* or *35S*::CA*‐MdMPK6‐15G‐GFP* constructs were transiently co‐expressed in tobacco leaves and protein extracts were immunoprecipitated with anti‐GFP beads. The precipitated proteins were then analysed by immunoblotting with an anti‐GFP antibody and an anti‐HA antibody. A band with the expected electrophoretic mobility of HA‐MdHY5 was detected in the anti‐GFP immuno‐precipitates from leaves expressing MdMPK6‐02G‐GFP, MdMPK6‐15G‐GFP, CA‐MdMPK6‐02G‐GFP, or CA‐MdMPK6‐15G‐GFP (Figure 3c). In contrast, HA‐MdHY5 was not detected in anti‐GFP immune‐precipitates from leaves harbouring the empty vector. Furthermore, a construct containing MdHY5 and the C‐terminus of yellow fluorescent protein (cYFP) was made, as well as constructs with the MdMPKs proteins and the N‐terminus of YFP (nYFP) to conduct bimolecular fluorescence complementation (BiFC) assays. Co‐transformation of MdMPK6‐02G‐nYFP and MdHY5‐cYFP; MdMPK6‐15G‐nYFP and MdHY5‐cYFP; CA‐MdMPK6‐02G‐nYFP and MdHY5‐cYFP; and CA‐MdMPK6‐15G‐nYFP and MdHY5‐cYFP proteins produced YFP fluorescence in tobacco cells, while no YFP fluorescence was detected in negative controls (Figure 3d). Meanwhile, no YFP fluorescence was observed after co‐transformation of MdMPK3s‐nYFP and MdHY5‐cYFP; MdMPK4s‐nYFP and MdHY5‐cYFP proteins in tobacco cells (Figure S3). These combined results indicate that MdMPK6 proteins interact with MdHY5 *in vitro* and *in vivo*.

To quantify the binding affinities of the MdMPK6 proteins to MdHY5, we compared the kinetic values of the interactions between MdMPK6‐02G‐MdHY5 and MdMPK6‐15G‐MdHY5 by biolayer interferometry (BLI). The KD value of the MdMPK6‐02G‐MdHY5 binding was higher than for the MdMPK6‐15G‐MdHY5 binding, suggesting that there is a relatively higher affinity between MdMPK6‐15G‐MdHY5 than between MdMPK6‐02G‐MdHY5. We deduced that MdMPK6‐15G was mainly responsible for the interaction with MdHY5 in the fruit (Figure 3e).

### Light‐induced phosphorylation of MdHY5 requires MdMPK6

**Figure 4:**
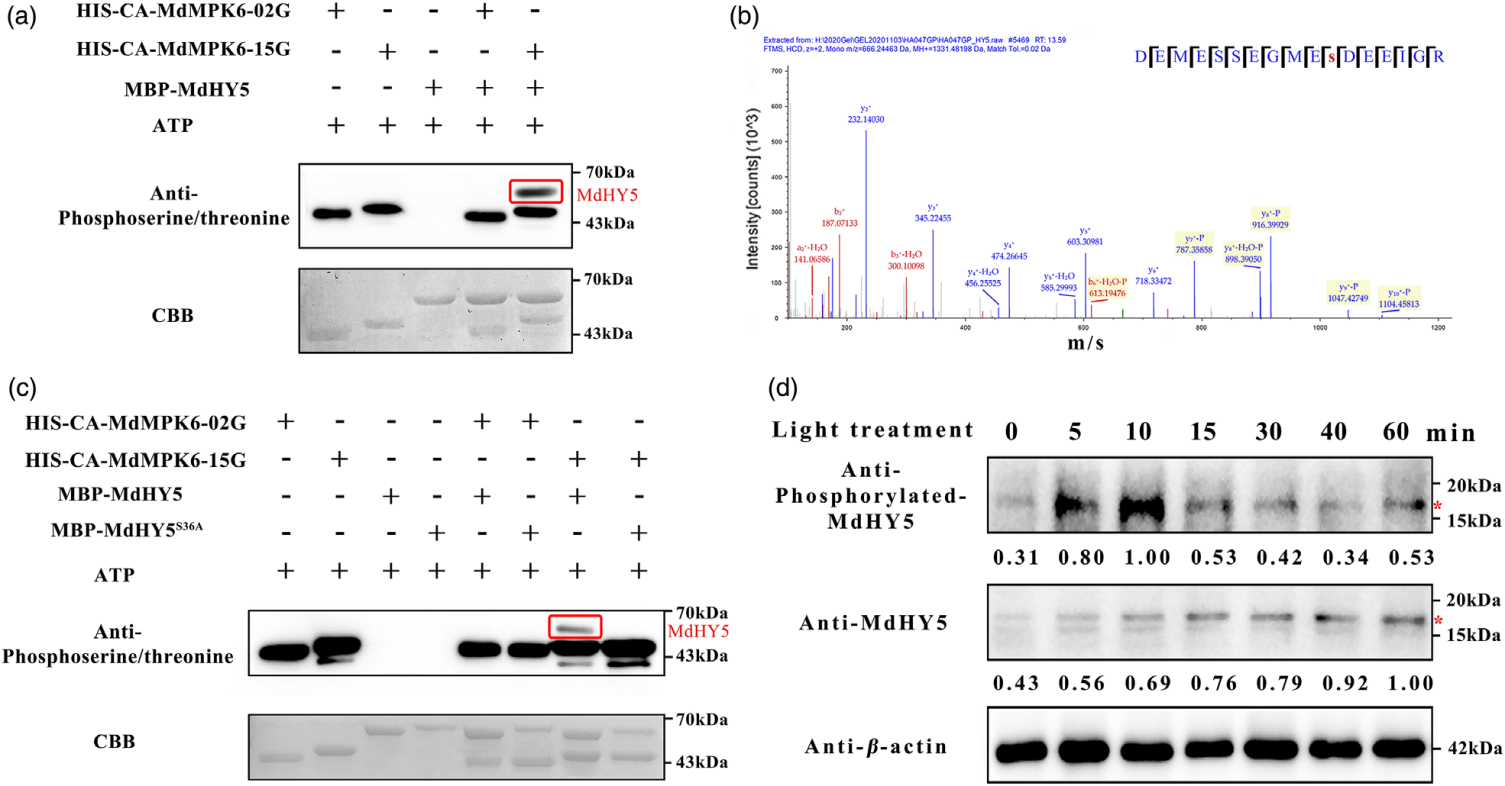
MdMPK6 phosphorylate MdHY5. (a) CA‐MdMPK6 proteins phosphorylate recombinant MdHY5. Myelin basic protein (MBP) was used as control substrate to confirm the kinase activity of CA‐MdMPK6. Autoradiography was conducted to visualize the phosphorylated MdHY5 protein. (b) Liquid chromatography–tandem mass spectrometry (LC–MS/MS) analysis showing that MdHY5 Ser‐36 is phosphorylated. The sequence of the doubly charged peptide ion at score 63.14, matches the MdHY5 EGMEsDEEIGR sequence. (c) Mutation of the putative MdHY5 phosphorylation site obviously reduces the phosphorylation of MdHY5 by CA‐MdMPK6‐15G. Recombinant MdHY5 and MdHY5^S36A^ were incubated with CA‐MdMPK6‐15G. Autoradiography was conducted to visualize the phosphorylated MdHY5 protein. Phosphorylated MdHY5 was marked with red box. (d) *In vivo* phosphorylation of MdHY5 debagged apple fruits was used to conduct light treatment at the indicated times. Phosphorylated MdHY5 was detected using an anti‐MdHY5 antibody in a Phosbind Acrylamide gel. Asterisk (*) represent specific MdHY5 band.

To test whether MdHY5 is an MdMPK6 substrate, an *in vitro* kinase assay was conducted. Proteins extracts were visualized using Coomassie Brilliant Blue (CBB) staining and the phosphorylated proteins were identified with an anti‐phosphoserine/threonine antiserum (Figure 4a). The results showed that MdHY5 is indeed phosphorylated by CA‐MdMPK6‐15G *in vitro*. Furthermore, in the presence of CA‐MdMPK6‐02G, 6×HIS MBP‐MdHY5 protein was not phosphorylated, suggesting that MdHY5 is not a MdMPK6‐02G substrate. The MdHY5 phosphorylation site was next determined by liquid chromatography‐tandem mass spectrometry (LC‐MS/MS) of TiO_2_ chromatography‐enriched MdHY5 phosphopeptides. Thirteen distinct phosphor‐peptides were detected that corresponded to the MdHY5 amino acid sequence (Figure 4b, Table S4). Previous studies showed that a single Ser‐36 site was the main phosphorylation site of HY5, a site that was also found in MdHY5 phosphopeptides (Hardtke *et al*., 2000). To validate the Ser‐36 site *in vivo*, it was mutated to Ala, and the phosphorylation level of MdHY5 and MdHY5^S36A^ recombinant proteins by CA‐MdMPK6‐15G were examined. The *in vitro* kinase assay also indicated that CA‐MdMPK6‐15G protein did not phosphorylate MdHY5^S36A^ (Figure 4c). To further confirm the MdHY5 phosphorylation site, we generated a phosphoantibody to the Ser‐36 site. Immunoreactive bands were observed using immunoblot analysis of extracts from fruit exposed to light (0, 5, 10, 15, 30, 40, and 60 min; Figure 4d), and the amino acid sequences of the immunoreactive bands were shown by LC–MS/MS to include the Ser‐36 site. MdHY5 phosphorylation increased after a 5 min light treatment, and the highest abundance of the phosphoprotein was observed after 10 min of light treatment, and the expression level of *MdHY5* was continuous increased with light treatment (Figure S4), which further suggested that MdHY5 is rapidly activated by light treatment (Figure 4d).

### MdMPK6 proteins participate in the accumulation of anthocyanin in apple peels

**Figure 5:**
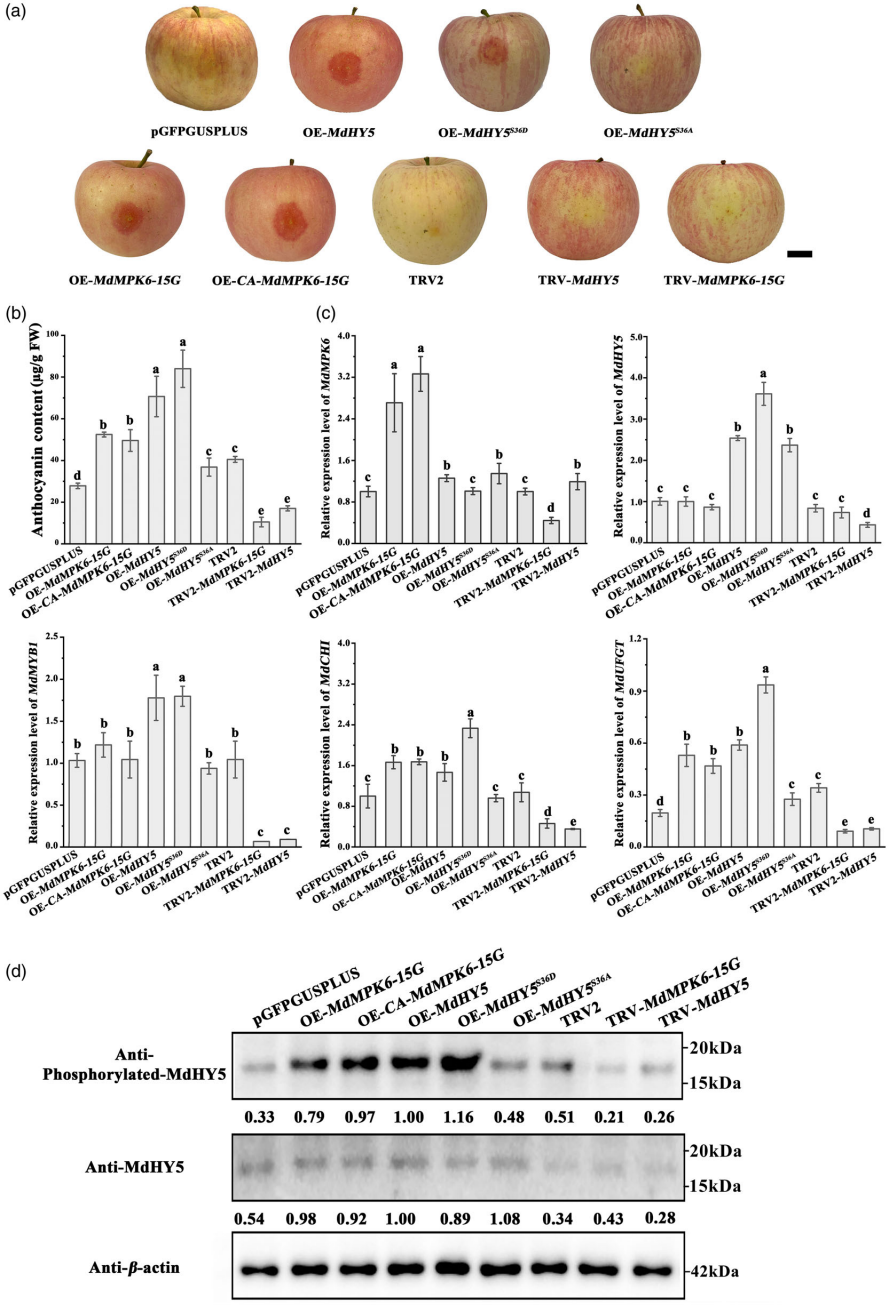
Overexpression or silencing of *MdMPK6‐15G*, CA‐*MdMPK6‐15G*, and *MdHY5* in the ‘Red Fuji’ apple cultivar. (a) The phenotype of infiltrated apple fruit was photographed 5 days post‐infiltration. Scale bar = 1 cm. (b) Anthocyanin content of apple fruit (μg/g fresh weight). (c) Transcription levels of *MdMPK6‐15G*, CA‐*MdMPK6‐15G*, and *MdHY5* and the anthocyanin biosynthesis genes, *MdCHI* and *MdUFGT* and anthocyanin biosynthesis regulator *MdMYB1* in infiltrated apple fruit were examined using RT–qPCR. (d) Immunoblot analysis showing accumulation of phosphorylated MdHY5 in infiltrated apple fruit under light conditions using an anti‐phosphorylated MdHY5 antibody. RT–qPCR, high‐performance liquid chromatography (HPLC) and immunoblot analysis were performed using three biological replicates. Data are means ± SD of three independent biological replicates. Different letters above the bars indicate significantly different values (*P* < 0.05), calculated using one‐way analysis of variance (ANOVA) followed by a Tukey's multiple range test.

To demonstrate that MdMPK6 is involved in light induced anthocyanin accumulation in apple peel, we used the pGUSPLUSPLUS vector to construct OE‐*MdMPK6‐15G*, OE‐CA‐*MdMPK6‐15G*, and used the TRV2 silencing vector to construct TRV2‐*MdMPK6. Agrobacterium* infiltration was used to transfer and integrate the vectors into apple peels. As shown in Figure 5a, OE‐*MdMPK6‐15G* and OE‐CA‐*MdMPK6‐15G* caused anthocyanin enrichment, and there was no significant anthocyanin accumulation in TRV2‐*MdMPK6*‐infiltrated apple fruit. HPLC results supported the phenotypes in infected apple fruit (Figure 5b). We also observed that the expression of anthocyanin biosynthesis genes *MdCHI* and *MdUFGT* and the anthocyanin biosynthesis regulator MdMYB1 was decreased in *MdMPK6*‐silenced fruit than in the control, and *MdCHI* and *MdUFGT* was induced in *MdMPK6‐*overexpressing apple fruit than in the control, partially confirming them to be MdHY5 targets (Figure 5c, Figure S5).

The above results demonstrate that MdMPK6 can interact with, and phosphorylate MdHY5 and so we hypothesized that the increase in anthocyanin levels in OE‐*MdMPK6* was accompanied by phosphorylation of MdHY5. To test this, we analysed the phosphorylation level of MdHY5 in the infiltrated apple fruit. Lower MdHY5 protein phosphorylation levels were detected in TRV2‐*MdMPK6* infiltrated apple, and higher MdHY5 protein phosphorylation levels were observed in OE‐*MdMPK6s* fruit (Figure 5d).

To explore whether MdMPK6 promoted light‐induced anthocyanin synthesis depends on the phosphorylation level of MdHY5, the sequences of MdHY5 (native form), a phosphomimic MdHY5^S36D^ form (Ser‐36 mutated to Asp), and a phosphodeficient MdHY5^S36A^ form (Ser‐36 mutated to Ala) were inserted into the pGFPGUSPLUS vector downstream from the *35S* promoter. Phenotypic analysis showed that high levels of expressions of *MdHY5* ^*S36D*^ and *MdHY5* in apple fruit promoted anthocyanin accumulation and red coloration around the injection sites when compared to the control infiltrated fruit. In contrast, expressing OE‐*MdHY5* ^*S36A*^ did not raise obviously anthocyanin accumulation (Figure 5a,b). When we examined the RNA abundance of the three MdHY5 target genes *MdMYB1*, *MdCHI* and *MdUFGT*, the highest levels were observed in *OE‐MdHY5* ^*S36D*^ infiltrated fruit (Figure 5c). Immunoblot analysis showed that a strong and a weaker band, respectively, of phosphorylated MdHY5 were detected in MdHY5^S36D^ and MdHY5^S36A^ infiltrated fruit (Figure 5d). These results suggested that MdMPK6 participates in anthocyanin biosynthesis by phosphorylating MdHY5, and that Ser‐36 is the predominantly phosphorylated residue.

### MdMPK6 proteins promote anthocyanin biosynthesis in apple calli

**Figure 6:**
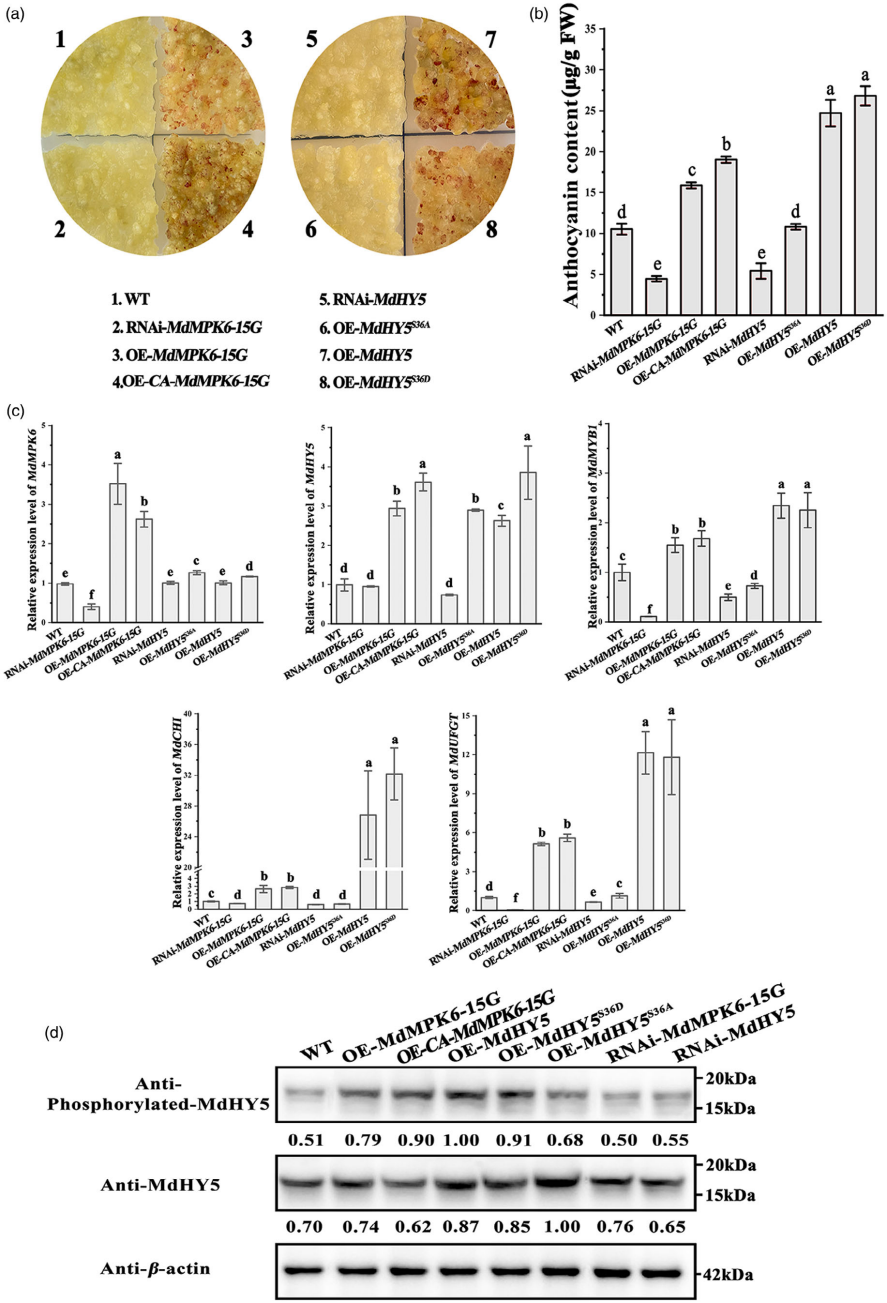
Overexpression or silencing of *MdMPK6‐15G*, CA‐*MdMPK6‐15G*, and *MdHY5* in apple calli. (a) Pigmented transgenic cells were formed in calli transformed with *MdMPK6‐15G*, CA‐*MdMPK6‐15G*, *MdHY5*, and *MdHY5* ^*S36D*^, but not in RNAi*‐MdMPK6*, RNAi*‐MdHY5*, and *MdHY5* ^*S36A*^‐overexpressing calli kept under constant high light at 16 °C for 12 days. (b) Anthocyanin content of transgenic apple calli (μg/g fresh weight). (c) Relative expression levels of *MdMPK6‐15G*, *MdHY5* and the anthocyanin biosynthesis genes, *MdCHI* and *MdUFGT* and anthocyanin biosynthesis regulator *MdMYB1* in apple calli, examined using RT–qPCR. (d) Immunoblot analysis showing accumulation of phosphorylated MdHY5 in apple calli kept under light using an anti‐phosphorylated MdHY5 antibody. RT–qPCR, high‐performance liquid chromatography (HPLC) and immunoblot analysis were performed using three biological replicates. Data are means ± SD of three independent biological replicates. Different letters above the bars indicate significantly different values (*P* < 0.05), calculated using one‐way analysis of variance (ANOVA) followed by a Tukey's multiple range test.

To further determine how MdMPK6 influences MdHY5‐promoted anthocyanin accumulation in apple, *MdMPK6* and *MdHY5* overexpression or silencing constructs were introduced into apple calli via *Agrobacterium*‐mediated transformation, and OE*‐MdMPK6‐15G*, OE‐CA‐*MdMPK6‐15G*, OE‐*MdHY5*, OE*‐MdHY5* ^*S36D*^, OE‐*MdHY5* ^*S36A*^, RNAi‐*MdMPK6*, and RNAi‐*MdHY5* transgenic calli was obtained. Anthocyanins accumulated in OE*‐MdMPK6*, OE‐CA‐*MdMPK6‐15G*, and OE*‐MdHY5* and OE‐*MdHY5* ^*S36D*^ apple calli but not in the control or other transgenic calli (Figure 6a), and no significant pigmentation was observed in OE‐*MdHY5* ^*S36A*^, RNAi‐*MdMPK6*, and RNAi‐*MdHY5* calli after light treatment. These results were confirmed by HPLC analysis of anthocyanin content (Figure 6b). Reverse transcription quantitative PCR analysis (RT–qPCR) showed that under light treatment, the MdMPK6‐15G, CA‐MdMPK6‐15G, MdHY5, and *MdHY5* ^*S36D*^‐overexpressing calli had much higher expression levels of the *MdMYB1*, *MdCHI*, and *MdUFGT* genes than the control (Figure 6c), while *MdMYB1*, *MdCHI*, and *MdUFGT* transcript levels were considerably lower in the OE‐*MdHY5* ^*S36A*^, RNAi‐*MdMPK6* and RNAi‐*MdHY5* lines than in the control (Figure 6c).

Subsequently, anti‐MdHY5 and anti‐phosphorylated‐MdHY5 antibodies were used for immunoblotting. The result showed that OE‐*MdMPK6*‐*15G*, OE‐CA‐*MdMPK6*‐*15G*, OE‐*MdHY5*, and OE‐*MdHY5* ^*S36D*^ transgenic apple calli exhibited stronger MdHY5 phosphorylation than the control and other transgenic calli. At the same time, weaker immunoreactive bands were observed in wild type, OE‐*MdHY5* ^*S36A*^ and RNAi‐*MdMPK6* transformed calli (Figure 6d), so we deduced that MdHY5 contributes to light‐induced‐promoted anthocyanin biosynthesis in an MdMPK6‐dependent manner.

### MdMPK6 prevent MdHY5 degradation under dark conditions

**Figure 7:**
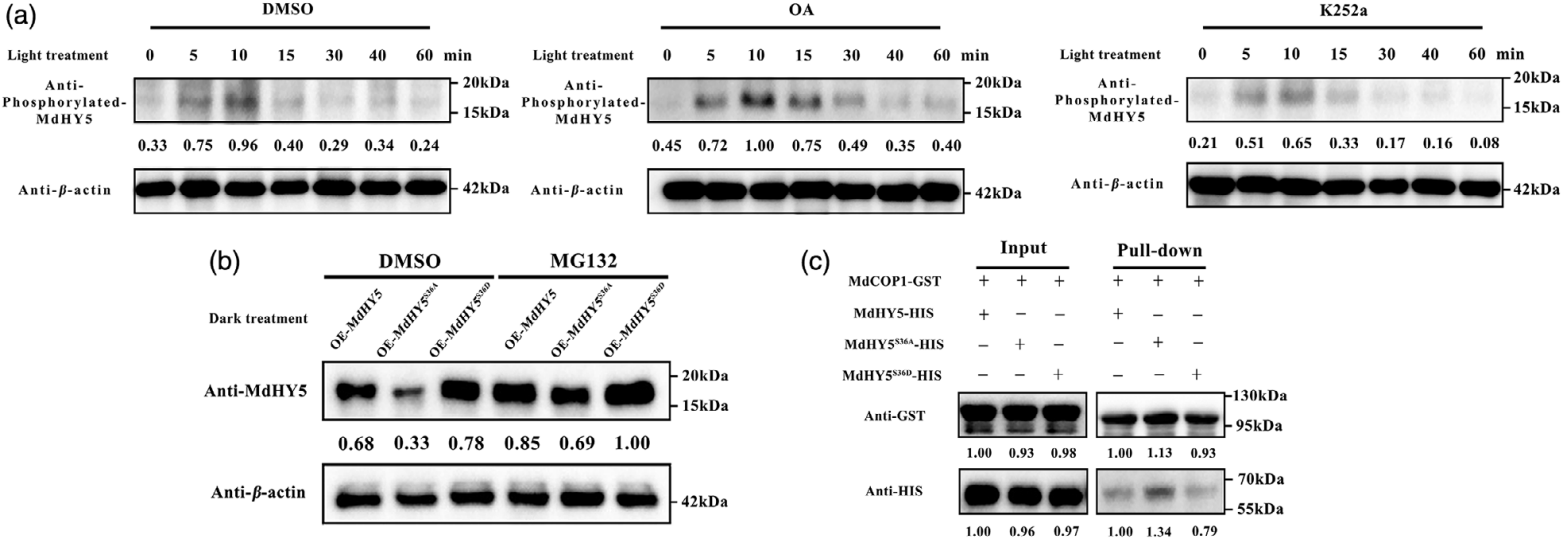
Accumulation of phosphorylated MdMPK6 and MdHY5 proteins in apple calli. (a) Apple calli were treated with a kinase inhibitor (K252a) or a phosphatase inhibitor (OA). Un‐treated calli at the indicated time points were used as a control. (b) Apple calli were treated with the 26S proteasome inhibitor (MG132) or DMSO (mock). *β‐*Actin was used as a protein loading control (bottom panel). (c) *In vitro* pull–down assay showing the interaction of MdCOP1 with MdHY5, MdHY5^S36A^, and MdHY5^S36D^. GST–MdCOP1 was immobilized on glutathione sepharose beads, incubated with MdHY5–HIS, MdHY5^S36A^–HIS, MdHY5^S36D^–HIS and subjected to immunoblot analysis with an anti‐GST or anti‐HIS antibody.

Next, we examined the phosphorylation level of MdHY5 in K252a‐ and OA‐treated apple calli. We observed that phosphorylated MdHY5 protein levels were substantially reduced after K252a treatment compared with the control (DMSO treatment), while they were significantly increased after OA treatment (Figure 7a). These results suggested that phosphorylation of MdHY5 is mediated by the MAPK cascade.

Previous studies showed that light‐mediated HY5 phosphorylation provides an added level of HY5 stability, and that unphosphorylated HY5 shows stronger interaction with COP1 in darkness, where it is also degraded via the proteasome pathway by the COP1 protein (Hardtke *et al*., 2000; Wang *et al*., 2021a). We evaluated the effect of MdMPK6‐mediated MdHY5 phosphorylation on MdHY5 stability using OE‐*MdHY5*, OE‐*MdHY5* ^*S36A*^, and OE‐*MdHY5* ^*S36D*^ transgenic apple calli treated with MG132, a 26S proteasome inhibitor under dark conditions. The results showed that MdHY5^S36D^ accumulated to high levels than that in MdHY5 and MdHY5^S36A^ in dark treated apple calli (Figure 7b). Our results indicated that MdMPK6‐mediated MdHY5 phosphorylation affects protein stability under dark conditions.

Finally, we performed *in vitro* pull–down assays to examine whether the phosphorylation of HY5 affects its interactions with MdCOP1. We observed that the MdHY5^S36A^ protein had a higher affinity for MdCOP1, whereas the MdHY5^S36D^ protein has a lower affinity to MdCOP1 than to MdHY5 (Figure 7c). Taken together, our results showed that phosphorylation alters the affinity of HY5 for COP1 and affects its accumulation.

### MdMPK6‐15G phosphorylate MdHY5 in the fruit ripening stage under light conditions

**Figure 8:**
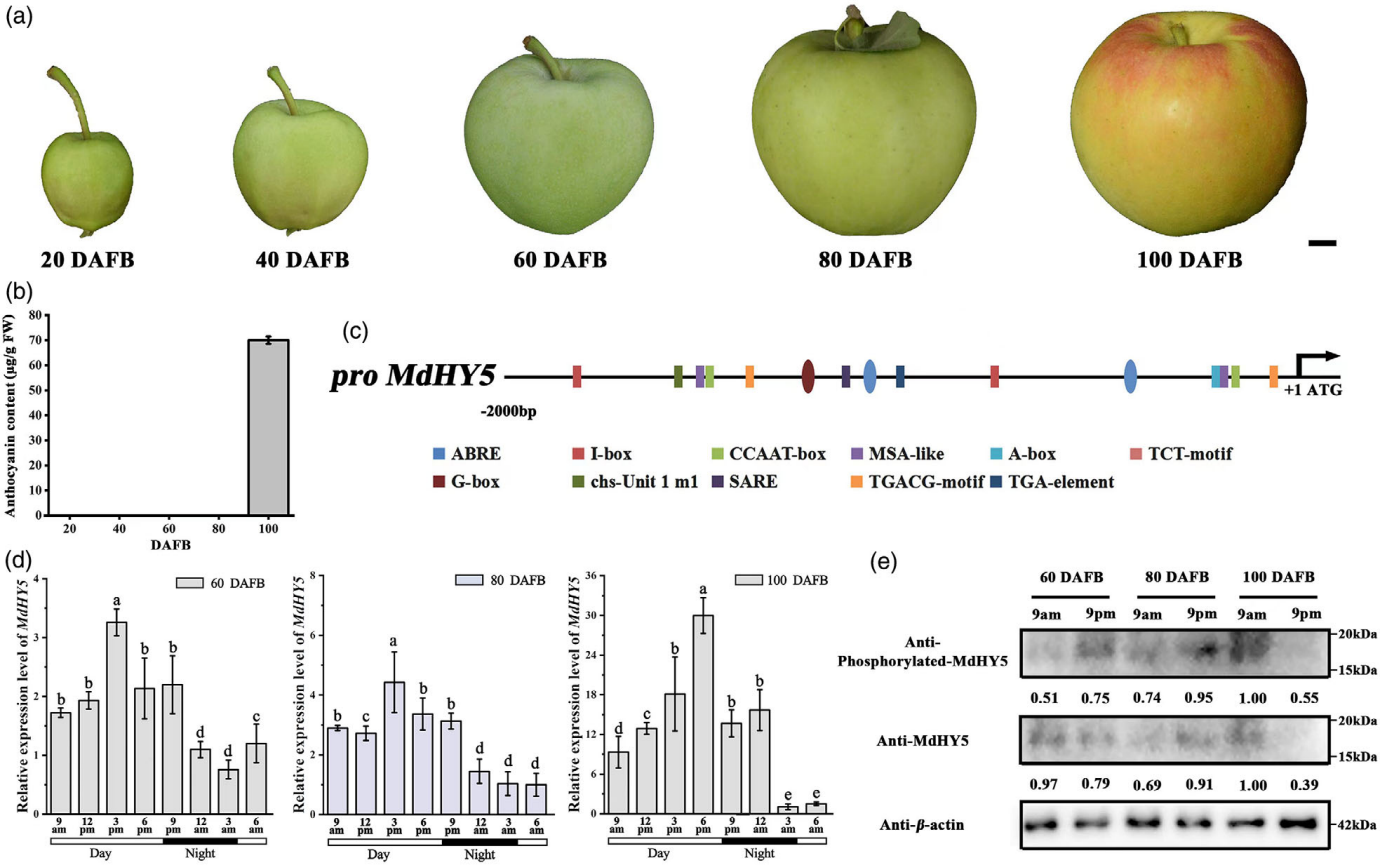
The phosphorylation of MdHY5 mainly occurs in the fruit ripening stage under light conditions. (a) Phenotypes (a) and anthocyanin contents (b) of ‘Gala’ fruit at different developmental stages. Scale bars = 1 cm. (c) Schematic illustration showing the distribution of light and methyl jasmonate (MeJA)‐responsive *cis*‐elements present in the MdHY5 promoter region using the PlantCARE database. (d) MdHY5 transcription levels in apple peels, determined by RT‐qPCR. Apple fruit at 60, 80, and 100 DAFB from plants grown under natural conditions were harvested in 3‐h intervals throughout a 24‐h period. The light period is indicated according to sunrise (represented by the white areas); darkness is represented by the dark areas. (e) Immunoblot analysis showing accumulation of MdHY5 and phosphorylated MdHY5 protein in infiltrated apple fruit between 9 am and 9 pm from 60 to 100 DAFB. Actin was used as a protein loading control. RT–qPCR, high‐pressure liquid chromatography (HPLC) and immunoblot analysis were performed using three biological replicates. Data are means ± SD of three independent biological replicates. Different letters above the bars indicate significantly different values (*P* < 0.05), calculated using one‐way analysis of variance (ANOVA) followed by a Tukey's multiple range test.

To further understand the biological function of MdMPK6‐15G phosphorylation of MdHY5 in apple fruit, we measured MdHY5 accumulation during fruit ripening. In ‘Royal Gala’ fruit, coloration was present in the fruit‐ripening stage (100 DAFB, days after full bloom), and HPLC results confirmed the presence of anthocyanins (Figure 8a,b), so we focused on the 60, 80, and 100 DAFB stages for subsequent analysis.

To characterize *MdHY5* expression, we analysed the *MdHY5* promoter region up to 2000 bp upstream from the translation start site using the PlantCARE database. Several light‐responsive elements were identified in the *MdHY5* promoter (including a chs‐Unit 1 m1; I‐box; G‐box), and we also identified a TGACG‐motif involved in methyl jasmonate (MeJA) responses, which suggested that *MdHY5* may participate in fruit ripening (Figure 8c). Next, we determined *MdHY5* transcript levels in 3‐h intervals over a 24‐h period at 60, 80, and 100 DAFB. *MdHY5* transcript levels exhibited a diurnal expression pattern, with higher expression during the day and lower expression at night, reaching a peak at 3 or 6 pm (Figure 8d).

To determine whether MdMPK6‐15G‐phosphorylated MdHY5 protein was also present in diurnal pattern, we assessed the abundance of MdHY5 and the phosphorylated form in the samples collected at 9 am and 9 pm at the different developmental stages. Immunoblot analysis indicated a large increase in the accumulation of MdHY5 in the fruit ripening stage (100 DAFB), and a higher abundance at 9 am than at 9 pm. This corresponded to the pattern of MdHY5 transcript levels (Figure 8d). MdHY5 phosphorylation levels in 60 and 80 DAFB were higher at 9 pm than at 9 am, but higher abundance at 9 am than at 9 pm was observed in the fruit ripening stage (100 DAFB), as examined using anti‐Phos‐MdHY5 antibodies. Meanwhile, MdHY5 phosphorylation levels correspond to the MdHY5 protein abundance (Figure 8e). To summarize, these results indicated that MdMPK6‐15G phosphorylation of MdHY5 mainly occurred in the fruit ripening stage and that this promoted anthocyanin accumulation in the daytime.

### MdHY5 functions synergistically with MdMPK6 to modulate anthocyanin accumulation in apple fruit

**Figure 9:**
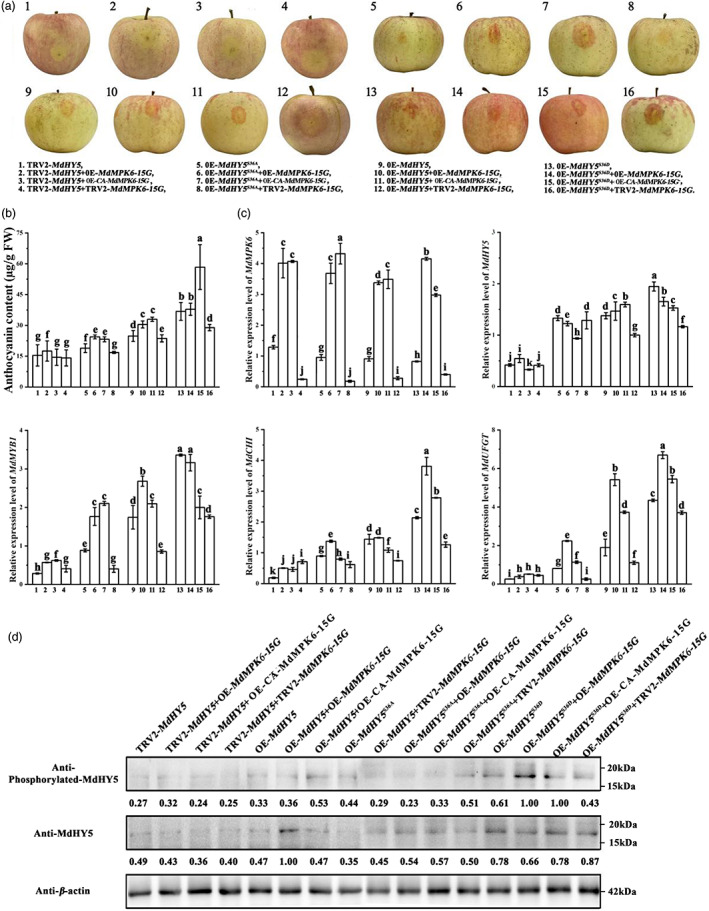
Co‐expressing MdMPK6 and MdHY5. (a) Apple fruit peel coloration around the injection sites. The full length *MdMPK6* and *MdHY5* cDNA sequences were cloned into the pIR vector for overexpression, whereas their cDNA fragments were inserted into the TRV vector for silencing. Scale bar = 1 cm. (b) Anthocyanin content in transformed apple fruit. (c) Relative expression levels of *MdMPK6*, *MdHY5* and anthocyanin‐related genes in transgenic calli as determined using RT–qPCR. The RT–qPCR and high‐pressure liquid chromatography (HPLC) analysis used to determine anthocyanin content were performed with three biological replicates. (d) Immunoblot analysis showing accumulation of phosphorylated MdHY5 in infiltrated apple fruit under light conditions using an anti‐phosphorylated MdHY5 antibody. RT–qPCR, HPLC, and immunoblot analysis were performed using three biological replicates. Data are means ± SD of three independent biological replicates. Different letters above the bars indicate significantly different values (*P* < 0.05), calculated using one‐way analysis of variance (ANOVA) followed by a Tukey's multiple range test.

To further test whether MdMPK6‐15G and MdHY5 work together to regulate anthocyanin biosynthesis during fruit coloration, we transiently overexpressed or silenced *MdMPK6‐15G* in the corresponding *MdHY5* transient expressed fruit. Elevated expression of *MdMPK6‐15G* and CA‐*MdMPK6‐15G* in *MdHY5*‐silenced fruit had no significant effect on anthocyanin accumulation, while overexpressing *MdMPK6‐15G* and CA‐*MdMPK6‐15G* in OE‐*MdHY5* and OE‐*MdHY5* ^*S36A*^ fruit significantly promoted anthocyanin accumulation compared to *MdHY5s*‐overexpressing fruit. When CA‐*MdMPK6‐15G* was overexpressed in OE‐*MdHY5* ^*S36D*^ fruit, the highest anthocyanin accumulation was observed. However, silencing of *MdMPK6‐15G* and CA‐*MdMPK6‐15G* significantly suppressed anthocyanin accumulation in *MdHY5*, *MdHY5* ^*S36A*^, and *MdHY5* ^*S36D*^ over‐expressing fruit (Figure 9a,b). RT–qPCR analysis showed that overexpressing *MdMPK6‐15G* and CA‐*MdMPK6‐15G* led to higher expression of biosynthesis and regulatory genes involved in anthocyanin accumulation in OE‐*MdHY5*, OE‐*MdHY5* ^*S36A*^, and OE‐*MdHY5* ^*S36D*^ fruit. *MdMPK6‐15G* and CA‐*MdMPK6‐15G* silencing in *MdHY5*, *MdHY5* ^*S36A*^, and *MdHY5* ^*S36D*^ over‐expressing fruit also suppressed the expression of anthocyanin biosynthesis and regulatory genes, although to a lesser degree (Figure 9c). These results indicated that the function of MdHY5 in regulating anthocyanin biosynthesis is dependent on its phosphorylation by MdMPK6.

**Figure 10:**
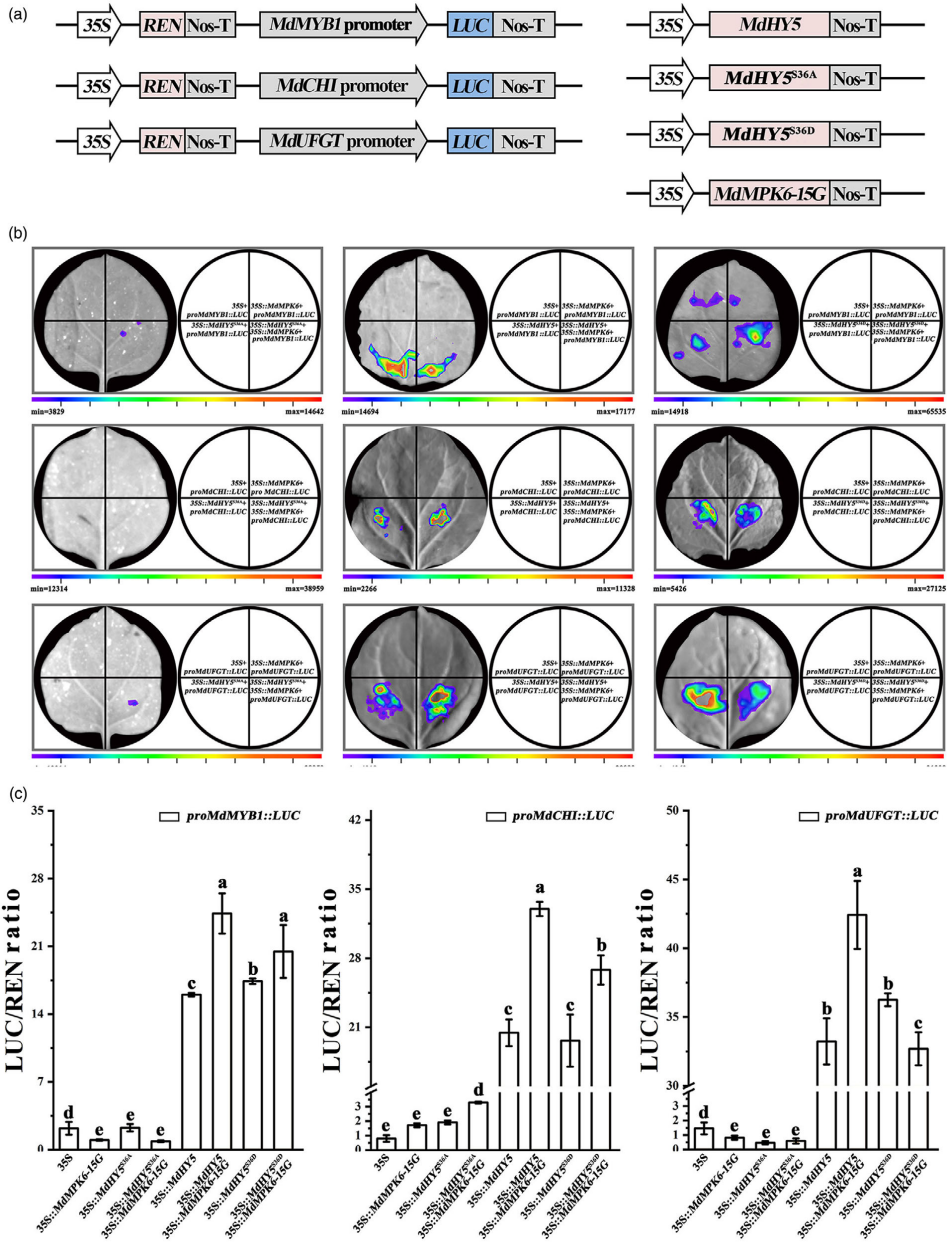
Dual luciferase assays in tobacco leaves were conducted to detect *in vivo* interactions between MdMPK6 proteins and MdHY5 and the anthocyanin biosynthesis gene promoters. (a) Schematic representation of the Luc reporter vector containing the promoters of *proMdCHI*, *proMdUFGT*, and *proMdMYB1* and the effector vector containing MdMPK6 and MdHY5. (b) Transient expression assays showing that co‐expressing MdMPK6 and MdHY5 proteins promotes the expression of anthocyanin related genes. Representative images of *Nicotiana benthamiana* leaves 72 h after infiltration are shown. (c) Relative LUC activity from transient expression analysis of the *MdMYB1*, *MdCHI*, and *MdUFGT* gene promoters co‐infiltrated with a plasmid containing genes for MdHY5 fused to the *35S promoter*.

Furthermore, to explore the MdMPK6 effect on MdHY5 function to regulate anthocyanin‐related gene expression, dual‐luciferase (LUC) analysis was used to detect the expression of LUC driven by the *MdMYB1*, *MdCHI*, and *MdUFGT* promoters. Different MdHY5 and MdMPK6‐15G effector constructs were expressed under control of the *35S* promoter, and co‐transformed with *proMdCHI*, *proMdUFGT* or *proMdMYB1:: GUS* reporter constructs and the internal control (*35S::LUC*) into *N. benthamiana* leaves (Figure 10). We observed that MdHY5 activated the expression of *MdMYB1*, *MdCHI*, and *MdUFGT*, and LUC activities for the *MdMYB1*, *MdCHI*, and *MdUFGT* promoters were significantly greater when *MdMPK6‐15G* and *MdHY5* were co‐expressed. MdHY5 and MdHY5^S36D^ displayed similar transcriptional activation ability, whereas MdHY5^S36A^ did not cause enhanced transcriptional activation of the anthocyanin related gene promoters.

## Discussion

Fruit coloration is controlled by the concerted action of various signalling pathways that integrate information regarding environmental conditions with that from developmental and metabolic cues (Field *et al*., 2001). Among these signals, light is a key factor in triggering anthocyanin biosynthesis (Kami *et al*., 2010). Physiological and biochemical studies have demonstrated that light‐induced fruit coloration is regulated by several MYB TFs in apple fruit. The apple MdMYB10/MdMYB1/MdMYBA TFs are thought to be activated by light and promote anthocyanin accumulation by activating the transcription of anthocyanin biosynthesis genes (Ban *et al*., 2007; Espley *et al*., 2007; Takos *et al*., 2006). Although a light‐induced anthocyanin biosynthesis mechanism has long been suggested, the identification of upstream regulatory components has been limited.

Plants have evolved four classes of photoreceptors to monitor the surrounding light conditions: red/far‐red light‐sensing phytochromes, blue/ultraviolet (UV) A light‐sensing cryptochromes and phototropins, and UVB light‐sensing UVR8 (Chen et al., 2004; Paik & Huq, 2019). Notably, all the light signals perceived by different photoreceptors converge on the central downstream transcription factor elongated hypocotyl 5 (HY5). In *A. thaliana*, more than 60% of early‐induced genes by phyA or phyB are HY5 direct targets, which is consistent with the notion that HY5 is one of the highest ranking regulators in the hierarchy of the transcriptional cascade that control diverse programs such as photomorphogenesis, nutrient signalling, and abiotic and biotic stress responses (Gangappa and Botto, 2016). Several studies have reported that HY5 is involved in the regulation of anthocyanin biosynthesis (An *et al*., 2017; Jiang *et al*., 2016; Shin *et al.*, 2013). In tomato, SlHY5 regulates light‐induced anthocyanin accumulation by directly binding to the promoters of anthocyanin biosynthesis genes, such as *CHS1*, *CHS2*, and *DFR* (Wang et al., 2021b). In addition, loss of function of tomato *HY5* (*SlHY5*) was reported to impair pigment accumulation, and a chromatin immunoprecipitation assay showed that anthocyanin biosynthesis genes are direct targets of SlHY5 (Wang *et al*., 2021a). In apple fruit, MdHY5 induces anthocyanin accumulation and positively regulates both its own transcription and that of MdMYB10 by binding to E‐box and G‐box motifs, respectively (An *et al*., 2017). We, therefore, deduced that HY5 is functionally located upstream from MdMYB10 and is a hub TF in anthocyanin biosynthesis transcriptional networks. Here, we confirmed that MdHY5 promoted light‐induced anthocyanin biosynthesis by overexpressing it into apple calli and fruit. Furthermore, yeast one‐hybrid (Y1H) assays and transient expression results showed that MdHY5 can bind to the promoters of the anthocyanin related genes *MdCHI*, *MdUFGT*, *MdMYB10*, and activate their transcription. Together, these results suggest that MdHY5 is located upstream of the anthocyanin biosynthesis transcription regulatory pathway and mediates light induced anthocyanin accumulation in apple fruit.

In a previous study, HY5 was found to be regulated by phosphorylation of the serine 36 residue (Hardtke *et al*., 2000). The unphosphorylated form of HY5 interacted more strongly with COP1 and was preferentially degraded under dark conditions, while it became more abundant in light‐grown seedlings (Hardtke *et al*., 2000), which suggested that phosphorylation may be essential for the activity of HY5 during light induced developmental processes. Recently, a study further confirmed that SPA proteins can directly phosphorylate HY5 and fine‐tune its stability and activity in regulating photomorphogenesis (Wang *et al*., 2021a). We hypothesized that a similar regulatory mechanism might be present in apple fruit. Our results showed that a light‐induced protein kinase, MdMPK6, phosphorylates MdHY5 under light treatment to promote its transcriptional activity in apple fruit. MdMPK6 overexpression in apple fruit and calli revealed that MdMPK6 enhances anthocyanin accumulation and induces *MdHY5* protein activity in response to light. Previous results showed that Ser‐36 is the phosphorylation site in *A. thaliana* (Hardtke *et al*., 2000), and the same was true for MdMPK6 phosphorylation of MdHY5 in apple. Phosphor‐inactive and mimic analyses indicate that MdHY5 activity in light‐induced anthocyanin accumulation is enhanced when MdHY5 is mutated to MdHY5^S36D^, and abolished when MdHY5 is mutated to MdHY5^S36A^. We conclude that MdMPK6‐mediated MdHY5 phosphorylation of Ser‐36 induces anthocyanin accumulation in fruit. We also noticed that MdMPK6 overexpression in apple promote the transcription level of MdHY5. Previously studies showed that HY5 has also been reported to feed back to promote its own transcription (Abbas *et al*., 2014; Binkert *et al*., 2014; Burko *et al*., 2020). So, we deduced that massive expression of MdMPK6 promoted the protein activity of MdHY5, and *MdHY5* promote its own expression.

Phosphorylation or ubiquitination of TFs provides a versatile means to regulate protein activity during plant development, and can enhance or suppress biological processes (Hunter, 2007). For example, in apple, the MdbHLH3 TF is phosphorylated by the protein kinase MdHXK1 in response to glucose signals, and ubiquitinated by high‐glucose‐inhibited U‐box‐type E3 ubiquitin ligase MdPUB29, to regulate ethylene biosynthesis and hence fruit quality (Hu *et al*., 2016, 2019). The HY5 ubiquitination mechanism has been well studied during plant photomorphogenesis. Here, HY5 stability is directly proportional to increased light intensity and, in darkness, its turnover is mainly regulated by the CONSTITUTIVE PHOTOMORPHOGENIC 1 (COPI)‐mediated 26S proteasome pathway (Osterlund *et al*., 2000). A recent study also showed that HY5 phosphorylation results in lower binding affinity to COP1 and SPA1, which reduces HY5 ubiquitination and, in turn, stabilizes HY5 proteins in the dark (Wang *et al*., 2021a). Here, a similar regulatory mechanism involving MdHY5 was uncovered in apple fruit. MdHY5, as a major light responsive regulator, can be ubiquitinated by MdCOP1 and phosphorylated by MdMPK6 to coordinate its own protein abundance, leading to the regulation of anthocyanin. Our previous study found that phosphorylation of the anthocyanin biosynthesis regulator MdMYB1 by MdMPK4 inhibits the MdCOP1–MdMYB1 interaction and prevents ubiquitination of MdMYB1 under light conditions (Yang *et al*., 2021). Thus, phosphorylation or ubiquitination provide an adaptable mechanism in light‐mediated signalling and facilitate the accumulation of anthocyanin biosynthesis regulators, thereby enhancing anthocyanin accumulation when fruit are exposed to light.

In a previous study, we found two *MdMPK4* genes in the apple genome, indicating that the MAPK gene family has expanded in apple (Yang *et al*., 2021). *MdMPK4‐06G* was more responsive to light treatment and catalysed phosphorylation of MdMYB1 in fruit, thereby modulating anthocyanin biosynthesis (Yang *et al*., 2021). *MdMPK4‐14G* can be activated by exposure to darkness and is involved in the dark‐induced degreening of fruit peels via MdERF17 phosphorylation (Wang *et al*., 2022). MdMPK4 proteins thus act as molecular bridges in both chlorophyll degradation and anthocyanin accumulation during apple fruit ripening. Two *MdMPK6* proteins were also identified in the apple genome, and the binding ability of MdMPK6‐15G to MdHY5 was found to be higher than that of MdMPK6‐02G to MdHY5. Moreover, only MdMPK6‐15G was found to phosphorylate MdHY5, so we hypothesized that, as with MdMPK4 proteins, the two MdMPK6 proteins have different functions, with *MdMPK6‐02G* participating in photomorphogenesis by phosphorylating other proteins. Future studies will elucidate the functions of the two *MdMPK6* copies.

**Figure 11:**
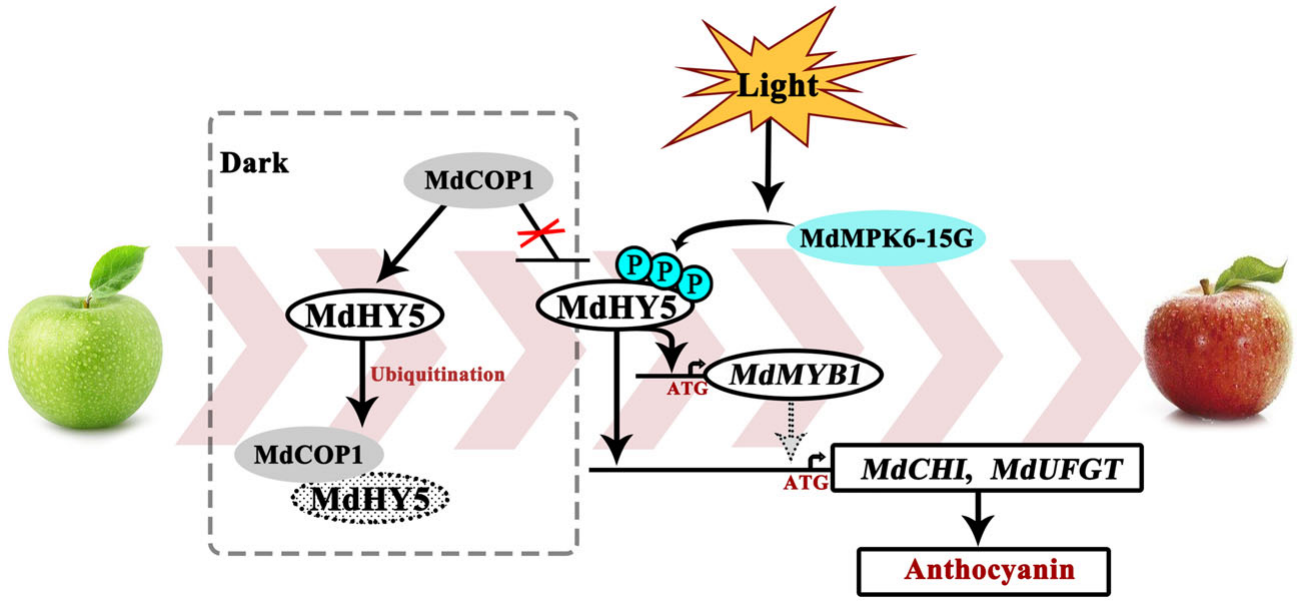
Model of MdMPK6 phosphorylation of MdHY5 mediates anthocyanin accumulation in apple fruit. MdMPK6 is activated by light treatment and interacts with and phosphorylates MdHY5. Phosphorylation of MdHY5 increases protein stability and prevent its degradation by MdCOP1, and promotes MdHY5 activation of anthocyanin‐related genes and anthocyanin accumulation in apple fruit.

In summary, our data reveal MdHY5‐mediated regulatory mechanism for promoting light induced anthocyanin accumulation, wherein MdMPK6 phosphorylates MdHY5 (Figure 11). During exposure of apple fruit to light, MdMPK6‐15G phosphorylates MdHY5 at Ser‐36 to activate its transcriptional activity. Phosphorylation of MdHY5 prevents it degradation by MdCOP1 and so increases its stability. These results suggest potential strategies for the biotechnological fine‐tuning of anthocyanin accumulation during light‐induced anthocyanin accumulation, and for guiding traditional breeding programs in creating new cultivars with improved fruit coloration.

## Methods

### Plant materials

Bagged apple (*Malus domestica* cv. ‘Red Fuji’) fruit were harvested 160 days after full bloom (DAFB) from the Germplasm Resource Nursery of *Malus* in Shunyi District, Beijing University of Agriculture (116°E, 40°N). The bagged fruits were placed in a dark incubator for 24 h at 23 °C to equilibrate the conditions. For light treatment, the harvested apples were transferred under low light from their bags to a white light incubator (390–780 nm, 215 μmol/m^2^/s^1^, 23 °C). Fruit peels were collected and frozen in liquid nitrogen for later analysis, which was conducted in three biological replicates. Apple calli were induced from young embryos of the ‘Orin’ apple cultivar (*M. domestica* Borkh.). Calli were sub‐cultured in MS medium containing 0.5 mg/L indole‐3‐acetic acid (IAA) and 1.5 mg/L 6‐benzylaminopurine (6‐BA) at 25 °C in the dark. The apple calli were sub‐cultured three times at 15‐day intervals before being used for genetic transformation and other assays (Li *et al*., 2012). Light treatment of debagged apple fruit (160 DAFB, before full ripeness, immediately after 1 day of dark treatment) and apple calli were performed by placing the samples under white (390 nm–780 nm, 12 000 lux) light. *Nicotiana benthamiana* was grown in a greenhouse at 25 °C under a 16 h day, 8 h night cycle.

### Protein extraction, peptide digestion, and peptide fractionation

Proteins were extracted from the apple fruit peels (three biological replicates per treatment) and homogenized in liquid nitrogen. Specifically, 20 g of apple peel sample, corresponding to approximately 2 mg protein, were added to a lysis buffer (4% SDS, 100 mm Tris/HCl pH 7.6, 0.1 m dithiothreitol (DTT), 5 mm sodium fluoride, 50 mm β‐glycerophosphate, 1 mm sodium orthovanadate, supplemented with the HALT protease and phosphatase inhibitor cocktail (Thermo Fisher Scientific, San Jose, CA, 78441)). Two milligrams of protein extract were digested with endoproteinase Lys‐C followed by trypsin according to the MED–FASP method (Wiśniewski and Mann, 2012). The trypsin digestion was stopped by adding trifluoroacetic acid (TFA) to a final concentration of 1% (vol/vol) and placed on ice then reconstituted in a 2% acetonitrile (ACN), pH 10 (pH adjusted using an ammonia solution (Wako, 013‐23355)) and centrifuged at 10 000 ***g*** to clarify the mixture. The peptides were then separated by basic‐pH reverse‐phase liquid chromatography (basic pH RPLC) using an Agela Durasbell C18 column (150°A, 5 μm, 4.6 × 250 mm) and a RIGOL L‐3000 HPLC. A gradient of solvent A (2% ACN, pH 10) and solvent B (98% ACN, pH 10) was used to separate the peptides based on their hydrophobicity at pH 10, as in Ren *et al*. (2018).

### TiO_2_ phosphopeptide enrichment and LC–MS/MS analysis

Phosphopeptides were enriched using titanium dioxide (TiO_2_) (Sugiyama *et al*., 2007; Thingholm *et al*., 2006). Briefly, a homemade micro‐column (200 μL) with a small plug of C8 material was prepared as previously described (Thingholm *et al*., 2006). TiO_2_ beads in tubes and TiO_2_ micro‐columns packed on centrifugation adaptors (GL Sciences, Shinjuku‐ku, Tokyo, Japan, 5010‐21514) were individually equilibrated with 100 μl of loading buffer (70% ACN, 5% TFA, 20% lactic acid (Sigma‐Aldrich, St. Louis, Missouri, USA, L6661)). After centrifugation, supernatants containing the TiO_2_ beads were removed.

Enriched phosphopeptides were resuspended in 10 μL of 10% FA and subjected to online reverse phase nano LC–MS/MS analysis with 50% of sample loading using an Easy nLC 1000 (Thermo Fisher Scientific), coupled to a Q‐Exactive mass spectrometer (Thermo Fisher Scientific; Ren *et al*., 2018).

### Bioinformatics analysis

Raw files were processed with MaxQuant (version 1.3.0.5) and aligned to the apple (*Malus domestica*) reference genome (Velasco *et al*., 2010). Protein function was annotated using the Blast2GO software (http://www.blast2go.com/b2ghome). The phosphopeptides were centered around the phosphorylated amino acid residues and aligned with seven positions upstream and downstream from the phosphorylation sites included. All differentially phosphorylated proteins that were classified as identifiers were queried for PPIs in the STRING database version 10.5 (http://string‐db.org/cgi/input.pl).

### Cloning of the *MdMPK6* genes and RT‐qPCR analysis

‘Red Fuji’ apple peel cDNA was used as a template to clone the full‐length *MdMPK6* and *MdHY5* genes. Genomic DNA was isolated from fruit peel to analyse *MdHY5* promoter sequence. *Cis*‐elements were predicted using the PlantCARE database (http://bioinformatics.psb.ugent.be/webtools/plantcare/html/) and the primers are listed in Table S5. Sequence comparisons and analyses were conducted as previously described (Kumar *et al*., 2004).

Gene expression levels were analysed by RT–qPCR using the Premix Ex Taq™ kit (Probe qPCR) (TaKaRa, Ohtsu, Japan), and a Bio‐Rad CFX96 Real‐Time PCR System (BIO‐RAD, Hercules, CA), according to the manufacturer's instructions. *MdActin* (LOC103453508) and *AtActin* (AT3G46520) were used as the internal controls. The data were analysed using the internal controls and the 2^(−∆∆Ct)^ method (Livak and Schmittgen, 2001). The threshold cycle (Ct) value of *MdActin* was subtracted from that of the gene of interest to obtain a ΔCt value. The Ct value of control sample was subtracted from the ΔCt value to obtain a ΔΔCt value. The fold changes in expression level relative to the control were expressed as 2^(−∆∆Ct)^ (Livak and Schmittgen, 2001). The mean ± SD is calculated by taking the mean of three biological replicates and then taking the mean of the individual samples. Analysis of the fruit, leaves and calli samples was repeated with three biological replicates. All the primer and probe primer sequences are listed in Table S5.

### Anthocyanin quantification

The HPLC analysis was used to determine apple peel anthocyanin content. The extraction was conducted as previously described (Tian *et al*., 2017), and anthocyanins were quantified by measuring absorbance at 520 nm using a calibration curve based on a commercial cyanidin‐3‐*O*‐glucoside standard (Sigma, St Louis, MO). The anthocyanin concentration was expressed as μg/g of fresh weight (FW; Revilla and Ryan, 2000). All the samples were analysed using three biological replicates (extracted from the peels of three different apples, calli, leaves, or plants).

### Protein extraction and immunoblot analysis

Proteins from apple calli and apple peel were extracted as previously described (Yang *et al*., 2021). Protein content was determined using a Bradford assay (Bradford, 1976). Antibodies used were as follows: anti‐GST (GST (91G1) Rabbit mAb #2625; 1:2000 dilution), anti‐ HIS (HIS‐Tag (D3I1O) XP® Rabbit mAb #12698; 1:1000 dilution), anti‐HA (HA‐Tag (Abcom) Mouse Ab 1424; 1:1000 dilution), anti‐GFP (Abcom Mouse Ab 291; 1:1000 dilution), anti‐*β*‐ACTIN (*β*‐Actin (D6A8) Rabbit mAb #8457; 1:1000 dilution), peroxidase‐conjugated goat anti‐rabbit IgG antibody (Anti‐rabbit IgG, HRP‐linked antibody #7074; 1:3000 dilution; Cell Signalling Technology, Danvers, MA); anti‐MdHY5, and anti‐phosphorylated‐MdHY5 (1:2000 dilution; produced by Beijing QiWei YiCheng Tech Co., Ltd, Beijing, China).

### Y2H, BiFC, pull‐down, Co‐IP and BLI assays

For directed Y2H assays, the *MdHY5* coding sequence was cloned into the pGADT7 vector as an activation domain, and *MdMPK3s*, *MdMPK4s*, *MdMPK6s*; and the constitutively active forms CA‐*MdMPK3s*, CA‐*MdMPK4s*, and CA‐*MdMPK6s* were cloned into the pGBKT7 vector as baits. Assays were performed according to the manufacturer's instructions (Invitrogen, Carlsbad, CA).

The *in vivo* BiFC assay was performed as previously described (Chen *et al*., 2013). MdMPK6‐02G, MdMPK6‐15G, CA‐MdMPK6‐02G, CA‐MdMPK6‐15G, and MdHY5 full‐length sequences were amplified and separately cloned into the SPYNE and SPYCE plasmids.

MdHY5‐GST, MdHY5‐6×HIS, CA‐MdMPK6‐6×HIS, and MdMPK6–GST proteins were expressed in *Escherichia coli* for use in a GST pull–down assay. Co‐IP was performed as previously described (Fiil *et al*., 2008). MdHY5, MdMPK6, and CA–MdMPK6 were generated as C‐terminal fusion proteins with HA or GFP tags. The elution products were analysed by Western blot analysis (Li *et al*., 2017). BLI assays were conducted on an Octet RED 96 System, according to the manufacturer's protocol (Gu *et al*., 2016).

### Recombinant protein purification

To promote the solubility of MdHY5, the coding sequence was inserted into the pGEX4T‐2, pETM‐41 vector (TaKaRa, Ohtsu, Japan; Qing *et al*., 2004) for low‐temperature induction. pGEX4T‐2‐MdHY5 and pETM‐41‐MdHY5 were transformed into *E. coli* (Rosetta strain) for *in vitro* induction with 0.5 mm IPTG (isopropylthio‐*β*‐galactoside) overnight at 16 °C. The MdHY5 protein was purified on a GST–Trap, HIS–Trap column and an AKTA purifier automatic chromatograph (GE Healthcare, Bucks, UK). The *MdMPK6* coding sequence was introduced into the pGEX4T‐2 and pETM‐41 vectors and transformed into *E. coli* (Rosetta), and the bacterial cultures were induced with 0.5 mm IPTG overnight at 16 °C. The MdMPK6 protein was purified on GST–Trap and HIS–Trap columns with an AKTA purifier automatic chromatograph (GE Healthcare).

### Phosphorylated antibody preparation

The antibodies for anti‐phosphorylated‐MdHY5 and anti‐MdHY5 were prepared by Beijing QiWei YiCheng Tech Co., Ltd. First, phosphorylated peptides and nonphosphorylated peptides of DEMESSEGMESDEEIGR were synthesized to a purity of over 90%. The phosphorylated peptides and non‐phosphorylated peptides were coupled with KLH to immunize rabbits. After the sixth immunization, an antiserum sample was taken to test the serum titre, and after the eighth immunization, the antiserum samples were collected. Second, a nonphosphorylated antigen affinity purification column was used to purify the antiserum to obtain antibodies recognizing the nonphosphorylated peptides until the peak of antibodies reached a certain low level. After all these antibodies were removed, a phosphorylated antigen affinity purification column was used to purify the antiserum to obtain the antibody recognizing the phosphorylated peptides.

### *In vitro* phosphorylation assays and LC–MS/MS analysis

*In vitro* phosphorylation assays were performed as previously described (Andreasson *et al.*, 2005). For MS analysis, 2 μg of recombinant MdHY5 protein was mixed with 1 mg of recombinant CA‐MdMPK6‐02G or CA‐MdMPK6‐15G protein in kinase reaction buffer containing 1 mm ATP. Reaction conditions and related analysis methods were as previously described (Li *et al*., 2016). Phos‐tag analysis was performed as previously described (Yin *et al*., 2018).

### Construction of expression vectors and stable transformation of apple calli

Overexpression vectors were made by amplifying the full‐length *MdMPK6‐15G* and *MdHY5* sequences from ‘Red Fuji’ apple peel cDNA, using RT–PCR. CA‐*MdMPK6‐15G*, *MdHY5* ^*S36A*^ and *MdHY5* ^*S36D*^ were generated using a site‐directed mutagenesis kit (TaKaRa, Ohtsu, Japan) and confirmed by sequencing. The corresponding cDNA sequences (*MdMPK6‐15G*, CA‐*MdMPK6‐15G*, *MdHY5* ^*S36A*^, *MdHY5*, and *MdHY5* ^*S36D*^) were cloned into the pGFPGUSPLUS plant transformation vector (Vickers *et al*., 2007) under the control of the CaMV 35S promoter. The RNAi vector (pK7GWIWG2) was made by amplifying the *MdMPK6‐15G* (1221 bp) and MdHY5 (518 bp) DNA fragments from ‘Red Fuji’ apple peel cDNA (Zhao *et al*., 2019). The DNA fragments were cloned into the pDONR207 entry vector and then assembled into the pK7GWIWG2 destination vector using Gateway recombination cloning technology (Thermo Fisher Scientific, Wilmington, DE). The constructed vector was transformed into *Agrobacterium* (LBA4404), which was then used to infect the calli to obtain the silenced tissue. All primers are listed in Table S5. Transformation of ‘Orin’ calli was performed as previously described (Li *et al*., 2012). Three independent transgenic apple calli lines were obtained.

### Transient expression in apple fruit and tobacco leaves

Bagged ‘Red Fuji’ apple fruit were harvested 140 days after bloom for transient expression. TRV vectors and *Agrobacterium* were constructed as previously described (Zhang *et al*., 2016). The infiltration protocol and culturing methods for transient expression assays were adapted from previously described methods (Liu *et al*., 2002; Zhang *et al*., 2016). The infected apples (‘Red Fuji’) were placed at 23 °C for either 2 days (for overexpression) or 5 days (for silencing). Infiltration was repeated using three biological replicates.

Dual‐luciferase assays were used to analyse *trans*‐activation of target gene promoters by TFs as previously described (Hellens *et al*., 2005; Xiang *et al*., 2015). The full‐length MdHY5 sequence and the *MdMYB1*, *MdCHI*, and *MdUFGT* promoters were cloned into the pGreenII‐62‐SK and pGreenII‐0800‐LUC vectors, respectively, with the primers listed in Table S5. The recombinant plasmids were transformed into *Agrobacterium* strain GV3101. Cultures expressing TFs and promoters were mixed (10:1 v/v) and then infiltrated into tobacco (*N. benthamiana*) leaves (Hellens *et al*., 2005). Firefly luciferase and *Renilla* luciferase enzyme activities were tested using the Dual‐Luciferase Reporter Assay System (Promega, Madison, WI). The enzyme activity assay was carried out with three biological replicates.

### Treatment of apple calli with kinase inhibitor (K252a) and phosphatase inhibitor (OA)

To determine the effects of K252a (kinase inhibitor) and OA (phosphatase inhibitor) on MdMPK6‐MdHY5 phosphorylation, calli expressing MdMPK‐15G and MdHY5 were light‐treated and phosphorylation levels of the two proteins were determined. Stably transformed calli were transferred to MS medium supplemented with 10 μm K252a or 1 μm OA (K252a #12754; Okadaic Acid #5934, Cell Signalling Technology, Danvers, MA), and cultured for 3 days in the dark. Wild‐type calli were used for comparison. The calli were treated with strong light and proteins were extracted as described above for Western blot analysis. Apple fruit were sprayed with 10 μm K252a or 1 μm OA and then stored in a growth room at 22 °C under natural light conditions with a daylight period of 16 h for 3 days. Three biological replicates were analysed.

### Treatment of apple calli with MG132

Apple calli were placed under high light for 2 days, prior to MG132 treatment. Apple calli were placed in medium with MG132 at a concentration of 100 μm and an aqueous solution of DMSO was used as the control treatment (Liu *et al*., 2013). The treated apple calli were placed in the dark for 12 h, and then proteins were extracted as described above for Western blot analysis. The experiments were carried out with three biological replicates.

### Data analysis

All the data were analysed using one‐way ANOVA followed by Tukey's multiple range test to compare differences between the experimental data at *P* < 0.05. Origin95, Microsoft Excel 2016 and IBM SPSS Statistics 22 were used for analyses. Quantification of band intensity was performed using ImageJ (https://imagej.nih.gov/ij/).

## Conflicts of interest

The authors declare no competing interests of this work.

## Author contributions

J.T. designed the project; F.‐Y. X, J.‐W. S., Y.‐Y. S., and J.‐L. L. performed the experiments; J.Z., T.‐T.S., Y.‐C.Y., and J.T. analysed the data and performed the research; J.T. and T.W. wrote the manuscript. All the authors read and approved the final manuscript.

## Supporting information


**Figure S1** Alignment of MPK3, MPK4, and MPK6 amino acid sequences in different species. Asterisks represent identical amino acids.
**Figure S2** Y2H analysis of the interaction between MdMPK3 and MdMPK4 proteins and MdHY5.
**Figure S3** Negative control of BiFC assay. Co‐transformation of MdMPK3s and MdMPK4s with MdHY5 proteins into tobacco cells, respectively.
**Figure S4** Expression analysis of MdHY5 under light treatment. RT‐qPCR was performed using three biological replicates. Data are means ± SD of three independent biological replicates. Different letters above the bars indicate significantly different values (*P* < 0.05), calculated using one‐way analysis of variance (ANOVA) followed by the Tukey's multiple range test.
**Figure S5** Analysis of interaction between MdMYB1, MdCHI, and MdUFGT promoters and MdHY5 protein. (a) Cis‐element analysis of the MdMYB1, MdCHI, and MdUFGT promoters. (b) Yeast one‐hybrid assay of MdHY5 and the MdMYB1, MdCHI, and MdUFGT promoters.

**Table S1** Information associated with the identified peptides.
**Table S2** Information associated with the identified phosphorylated peptides.
**Table S3** List of differentially phosphorylated proteins (DPPs) in protein–protein interaction (PPI) networks.
**Table S4** Identification of protein modifications.
**Table S5** Primers used in this study.
