# Supplementary material for: MPK6‐mediated HY5 phosphorylation regulates light‐induced anthocyanin accumulation in apple fruit
Source: Plant Biotechnol J. 2022 Oct 27;21(2):283–301. doi: 10.1111/pbi.13941 (PMC9884024; doi:10.1111/pbi.13941)
Supplement: Supplementary file 1 — Figure S1 Alignment of MPK3, MPK4, and MPK6 amino acid sequences in different species. Asterisks represent identical amino acids. Figure S2 Y2H analysis of the interaction between MdMPK3 and MdMPK4 proteins and MdHY5. Figure S3 Negative control of BiFC assay. Co‐transformation of MdMPK3s and MdMPK4s with MdHY5 proteins into tobacco cells, respectively. Figure S4 Expression analysis of MdHY5 under light treatment. RT‐qPCR was performed using three biological replicates. Data are means ± SD of three independent biological replicates. Different letters above the bars indicate significantly different values (P < 0.05), calculated using one‐way analysis of variance (ANOVA) followed by the Tukey's multiple range test. Figure S5 Analysis of interaction between MdMYB1, MdCHI, and MdUFGT promoters and MdHY5 protein. (a) Cis‐element analysis of the MdMYB1, MdCHI, and MdUFGT promoters. (b) Yeast one‐hybrid assay of MdHY5 and the MdMYB1, MdCHI, and MdUFGT promoters. [file PBI-21-283-s002.doc]

**
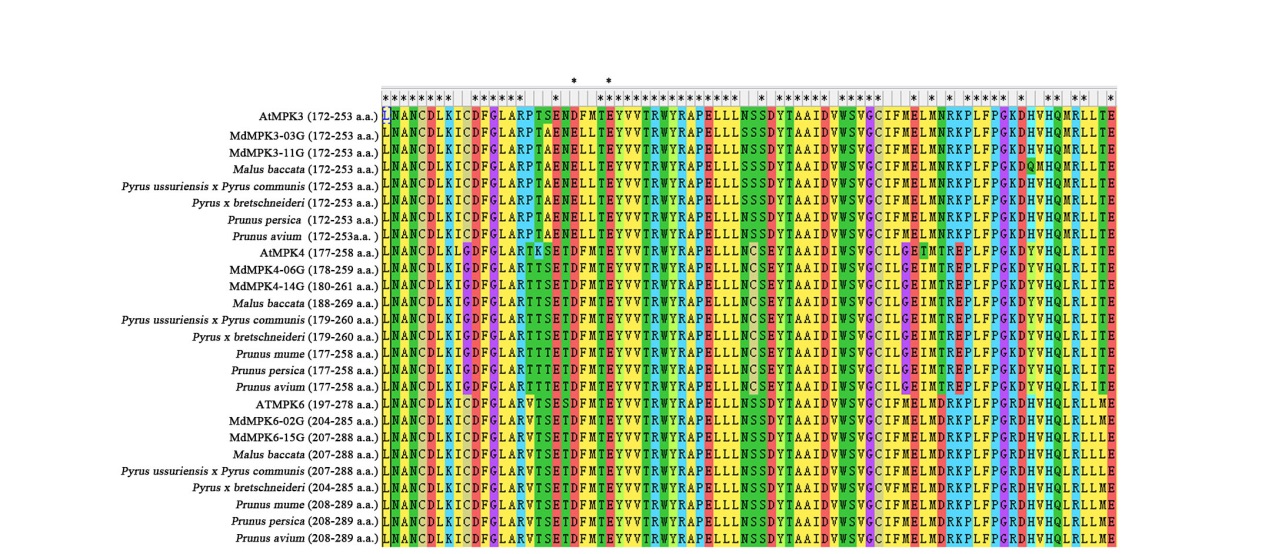
**

**Figure S1** Alignment of MPK3, MPK4 and MPK6 amino acid sequences in different species. Asterisks represent identical amino acids.


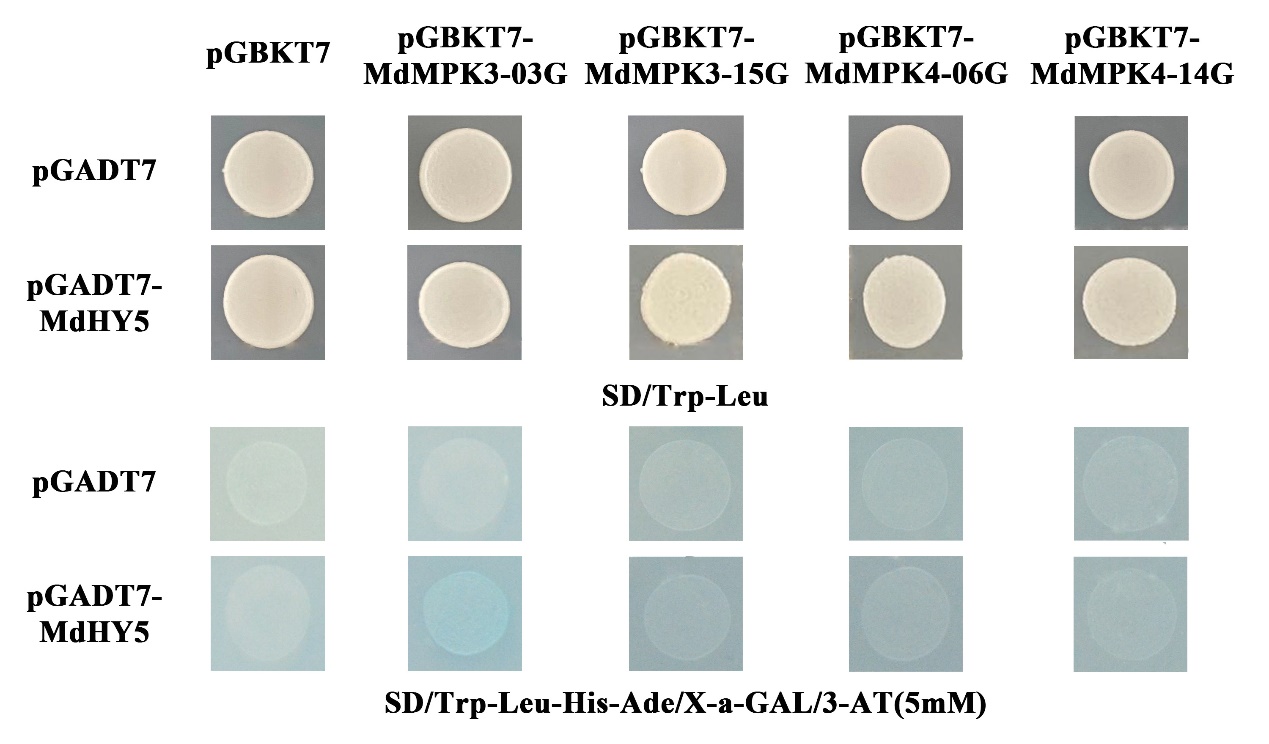


**Figure S2** Y2H analysis of the interaction between MdMPK3 and MdMPK4 proteins and MdHY5.

**
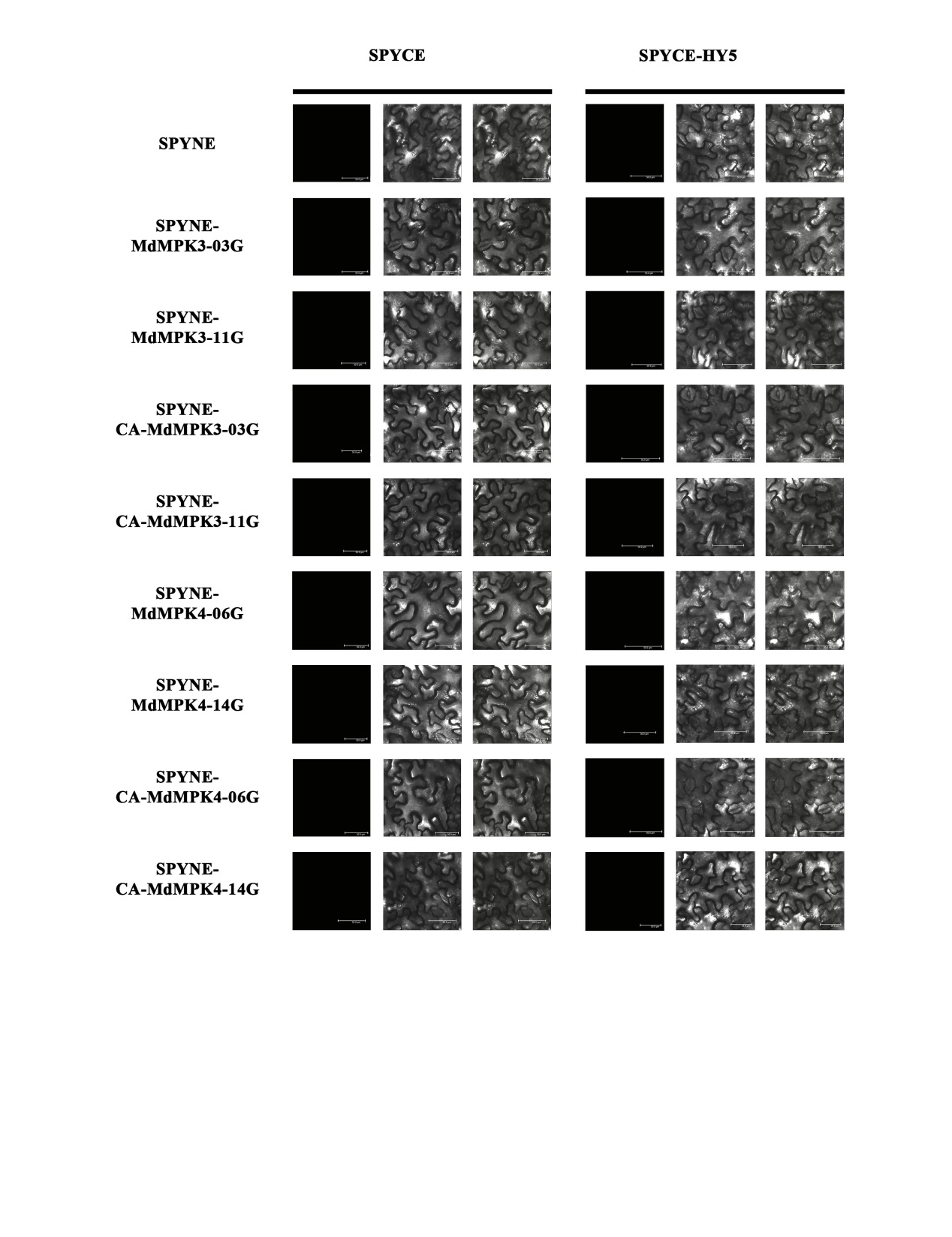
**

**Figure S3** Negative control of BiFC assay. Co-transformation of MdMPK3s and MdMPK4s with MdHY5 proteins into tobacco cells, respectively.


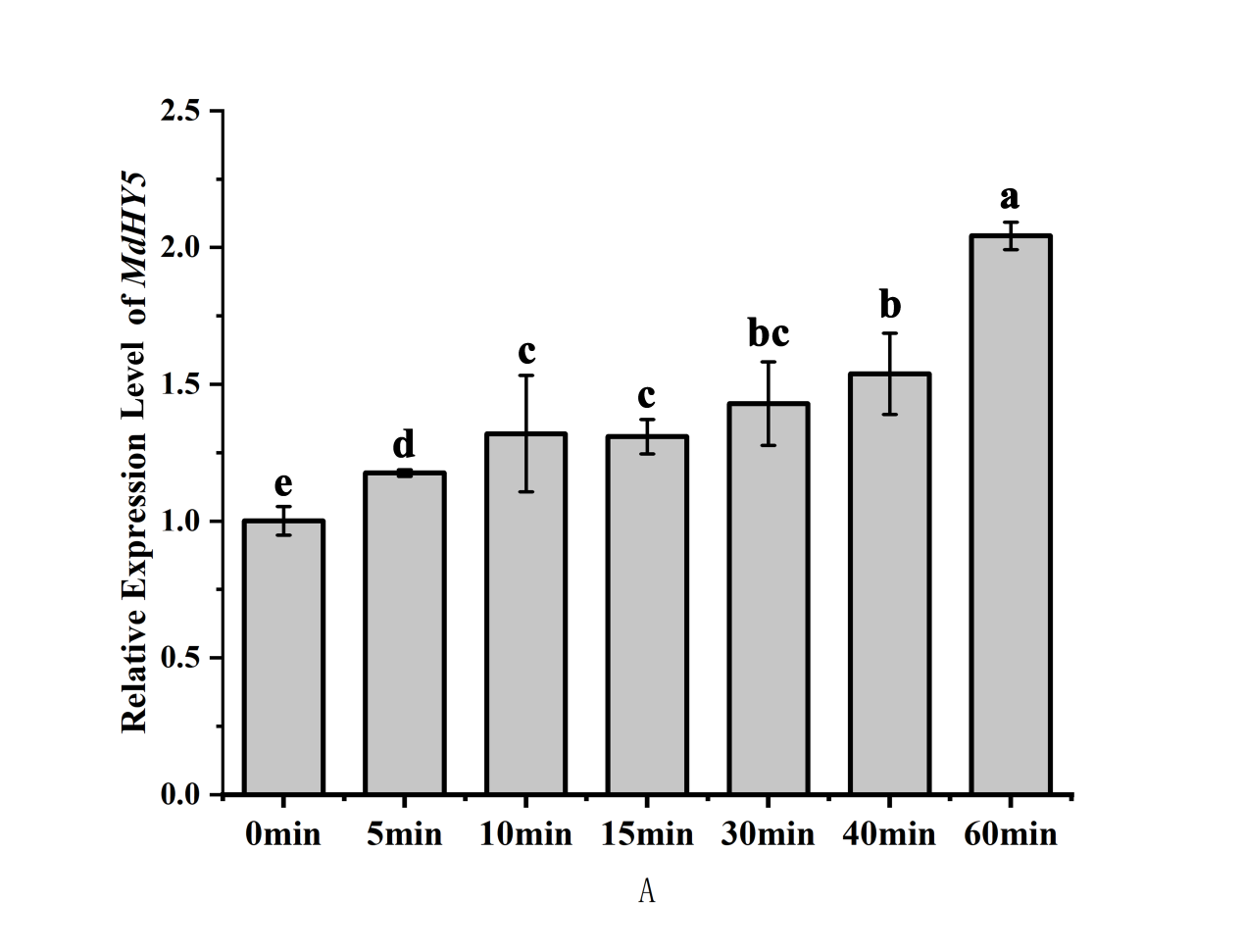


**Figure S4** Expression analysis of MdHY5 under light treatment. RT-qPCR was performed using three biological replicates. Data are means ± SD of three independent biological replicates. Different letters above the bars indicate significantly different values (P < 0.05), calculated using one-way analysis of variance (ANOVA) followed by a Tukey’s multiple range test.


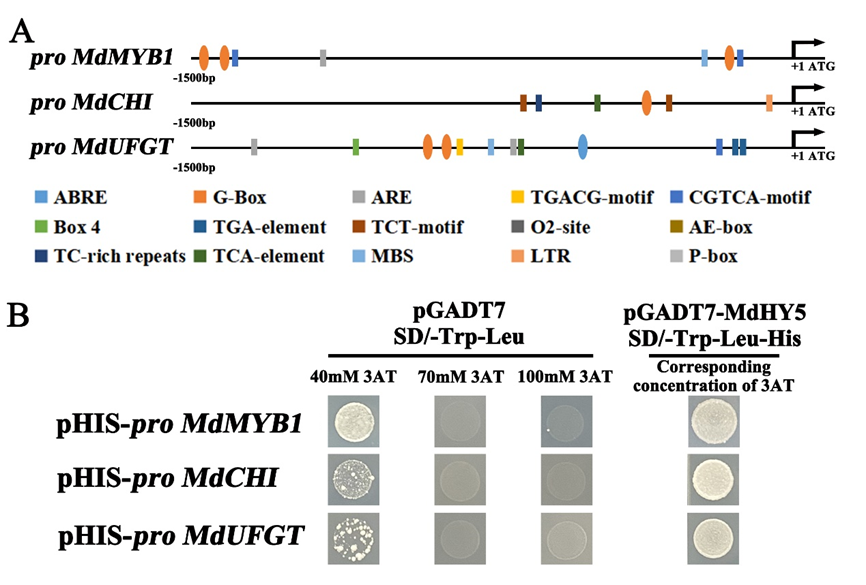


**Figure S5** Analysis of interaction between *MdMYB1*, *MdCHI* and *MdUFGT* promoters and MdHY5 protein. (a) *Cis*-element analysis of the *MdMYB1*, *MdCHI* and *MdUFGT* promoters. (b) Yeast one-hybrid assay of MdHY5 and the *MdMYB1*, *MdCHI* and *MdUFGT* promoters.
